# Design, Sustainable Processing and Nanoliposome Encapsulation of Red Grape Pomace Rich in Polyphenolic Compounds with Antioxidant Activity

**DOI:** 10.3390/molecules31010072

**Published:** 2025-12-24

**Authors:** Katarzyna Hałdys, Agnieszka Ciechanowska, Agnieszka Lewińska

**Affiliations:** 1 Department of Chemical Technology, Wroclaw University of Economics and Business, Komandorska 118/120, 53-345 Wroclaw, Poland; agnieszka.ciechanowska@ue.wroc.pl; 2 Faculty of Chemistry, University of Wroclaw, Joliot-Curie 14, 50-383 Wroclaw, Poland

**Keywords:** polyphenols, grape pomace, extraction, nanoliposomes, encapsulation

## Abstract

In this study, we aimed to investigate the potential of utilizing red grape pomace as a source of polyphenolic compounds in the growing, fragmented winemaking sector in Poland. For polyphenol extraction, we compared two methods: conventional extraction using water and alcohol solutions, and the supercritical CO_2_ technique with ethanol as a cosolvent. The conventional method yielded at least 30% more polyphenols compared to the advanced SC-CO_2_ technique. Experimentally chosen conditions, including a solvent composition of ethanol–water (1:1; *v*/*v*) containing 3% HCl, a liquid-to-solid ratio of 25:1 mL/g, and 2 min of ultrasound pretreatment and conventional extraction at a temperature of 30 °C over 4.5 h, enabled an extraction efficiency of 101 mg of total polyphenols per 1 g of raw material used, with an antioxidant capacity equivalent to 600 µmol of Trolox. According to HPLC analyses, the main components of the investigated biomass were epicatechin, anthocyanins and *p*-coumaric acid. The extract was encapsulated in liposomes, revealing no negative effect on their stability or aggregation under the conditions tested (21 days). The study suggests that conventional water–ethanol extraction can be a relatively safe and effective method for managing winemaking residuals, increasing the competitiveness of small producers through the production of high-value antioxidant additives.

## 1. Introduction

Antioxidants are molecules from both natural and synthetic sources that protect cells against oxidative stress. This process can be defined as an imbalance between the production of free radicals and the ability of the body to eliminate reactive species [1]. Antioxidants find application in multiple fields, such as medicine, the food industry and cosmetics, as well as additives that delay oxidation and material degradation during the processing and usage of polymers [2,3]. Natural antioxidants, primarily derived from plants, are gaining attention due to their safer profile and biocompatibility. Natural compounds with antioxidative properties include vitamin C, E, carotenoids and polyphenols [4].

Polyphenol extraction is commercially performed using conventional methods with aqueous and ethanolic solutions, which involve maceration, Soxhlet extraction and decoction [5]. To increase the efficiency, selectivity and automation of the methods, as well as to limit solvent consumption, novel technologies for polyphenol extraction are being explored, including ultrasound- or microwave-assisted extraction, deep eutectic solvent extraction, pressurized liquid extraction, supercritical fluid extraction (mostly with CO_2_), enzymatic extraction and pulsed electric field treatment [6]. However, the conventional methods, despite their limitations, are considered simpler and do not require expensive and complicated equipment.

Extracted polyphenolic compounds can be used in various branches of the food, pharmaceutical and cosmetic industries as antioxidants, enzyme inhibitors [7,8] or additives to new biodegradable materials [9,10,11]. Due to their key bioactive substances, polyphenolic extracts are susceptible to degradation and can also cause undesirable interactions with biological membranes. A solution to these problems may be their encapsulation in delivery systems, such as nanoemulsions [12,13], polymer carriers [14], solid lipid nanoparticles [15] for hydrophobic substances, and liposomes or multiple emulsions for hydrophilic substances. On the other hand, multiple emulsions and liposomes are best suited for hydrophilic substances. Liposomes are formed due to the tendency of phospholipids to aggregate, which results from their exceptionally favorable HLB values as amphiphiles and their molecular shape. This favors the formation of bilayer aggregates. Liposomal vesicles form by minimizing the total edge energy that occurs during the folding of a planar bilayer and forming closed structures. Generally, the following techniques are used to obtain liposomes: thin-lipid film hydration, sonication, ethanol injection, ether injection, detergent dialysis or reverse-phase evaporation. Depending on the technique used, liposomes of varying sizes and with varying numbers of layers can be obtained. Liposomes should be prepared at temperatures higher than the temperature of their main phase transitions, but should not exceed 45–50 °C. Most techniques allow the production of liposomes, but they are characterized by high heterogeneity; therefore, a liposome suspension is usually subjected to a process of standardization of their size using liposome dimensional calibration techniques. Methods for encapsulating active substances in liposomes can be broadly divided into passive and active methods. Passive encapsulation involves enclosing a substance in a hydrophobic or hydrophilic space and is the simplest and easiest to implement. It is somewhat spontaneous, depending solely on the phospholipid composition of the liposomes and the physicochemical nature of the encapsulated substance. Depending on the nature of the substance, it can be dissolved in lipids (hydrophobic) or added to the solution, hydrating a thin layer (hydrophilic). More advanced active techniques involve the use of additional factors, phenomena and chemical reactions. These include encapsulation along a pH or ion gradient, as well as the use of ionophores. Each of these techniques has its advantages and disadvantages, and the choice of production method depends on the type of ingredients and the potential application. Carriers not only serve a protective function but also enable the active substance to reach sites that it cannot reach by itself. Furthermore, encapsulation and targeted delivery not only increase therapeutic efficacy but also enhance cost-effectiveness. Carrier surfaces can also be chemically modified to increase their affinity for specific cellular or tissue targets.

According to the Sustainable Development Goals (SDGs) endorsed by the United Nations in 2015 [16], responsible consumption and production (SDG 12) follows the waste-to-resource trend within the circular economy model. In the promotion of sustainable development, a crucial role is played by the valorization of plant-derived biomass waste, including polyphenol-rich residuals from the processing of coffee, tea and various fruits and vegetables [16,17]. Among these raw materials, red grape pomace, the main residue generated from winemaking, is a valuable resource rich in bioactive compounds, including polyphenols and other antioxidants that can be recovered for alternative uses [18]. Besides high-value substances, the residues of the winemaking process include lignocellulose, a feedstock for further processing within the concept of biorefinery [19,20]. More importantly, the expanding wine industry, which produces around 9 million tonnes of waste annually, ensures abundant availability of grape pomace for valorization [18].

Climate change, consisting mainly of an increase in average temperatures during the growing season, may favor the development of wine production in regions previously of marginal importance in world wine production [21], with Poland as an example [22,23], especially its warmer southern area [24]. Polish grapes have a lower sugar content and higher acidity compared to the same varieties traditionally grown in countries with a warmer climate in southern Europe (e.g., Italy or Spain) [20,25].

The improving conditions for grapevine vegetation in Poland have led to significant growth of the wine production sector. According to data from KOWR (the National Support Centre for Agriculture, registry maintained since 2022), the number of entrepreneurs declaring wine production increased from 425 in 2022/2023 to 461 in 2023/2024 and 504 in 2024/2025, and as of 27 May 2025 there are 684 registered vineyards in Poland [26]. The development of the wine sector has consequently led to the generation of an increasing amount of waste which needs to be reused.

A literature review conducted to determine the optimal extraction conditions for polyphenols from grapes revealed a wide range of results and significant differences. Some general assumptions could be made, but the great diversity of the waste sources and differentiated growing conditions indicate the need for general optimization of the extraction process, starting with new waste biomass. The aim of the study was to optimize the first step of Polish grape waste management after wine production, following a biorefinery approach.

The waste biomass processing considered in this study includes biomass preparation (dehydration and grinding) and the influence of extraction factors, such as solvent composition, ultrasound assistance, time and temperature, and liquid-to-solid ratio (l/s). The conventional solvent method was compared with the industrial supercritical CO_2_ (SC-CO_2_) technique. Subsequently, water-soluble polyphenols were encapsulated in liposomes, and the physicochemical characterization of the obtained liposomes was analyzed. As a result, we present a simple and cost-effective route for potentially obtaining high-value products—antioxidative cosmetic additives sourced from the processing of grape pomace from Polish wineries. This approach provides a waste management process for small, developing winemakers with the potential for income diversification.

## 2. Results and Discussion

### 2.1. Selection of Parameters and Solid–Liquid Extraction Operations

#### 2.1.1. Solvent Composition

The choice of solvent is one of the most crucial steps in the design of polyphenol extraction. Since polyphenols are polyhydroxy compounds typically soluble in water, alcohols and aldehydes, the most commonly used extraction media are polar solvents such as water, acetone, methanol and ethanol. However, mono-component solvent systems are less efficient in extracting phenolic constituents than their mixtures, especially when aqueous solutions containing at least 50% water are used [27]. This is due to the diversified structures and polarity of various polyphenolic compounds. Consequently, their extraction requires both water and an organic solvent in the binary extraction medium. Thus, optimizing the composition of the extraction media is essential to achieve high process efficiency [27].

The selection of solvents based on sustainability and toxicity is also an important consideration. Water, the safest and greenest solvent, requires an organic cosolvent to increase efficiency. In turn, methanol is quite an efficient extractant, but along with acetone it is considered toxic [28]. Ethanol–water mixtures seem to be the most suitable solvents for extraction because of the different polarities of both solvents, the possibility of mixing them in any proportion and their acceptability for human consumption. Such ethanol–water extracts could be safely introduced into food products without the risk of unacceptable levels of hazardous solvent residues.

**Figure 1:**
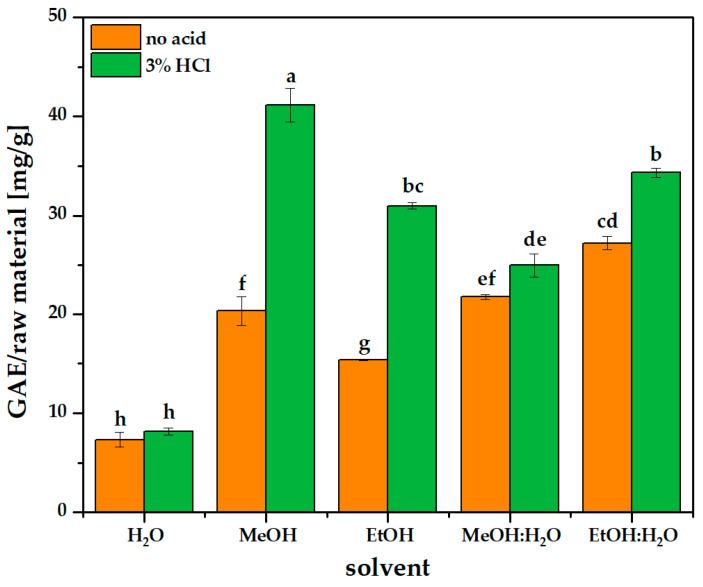
Polyphenol content in raw material extracted from oven-dried grape pomace depending on solvent composition. Extraction conditions: 30 °C, 20 h, l/s 50:1. Ratio of alcohol–water solution was 1:1 (*v*/*v*). Different lowercase letters above the bars indicate statistically significant differences (*p* ≤ 0.05) of the means (*n* = 3).

Consequently, water and ethanol were selected as safe and sustainable extractants, while methanol was selected as the solvent with the highest potential for polyphenol extraction. Studies on mono-component systems have confirmed that methanol has the highest extraction efficiency (total polyphenol content), achieving 20% and 60% higher results than the extraction efficiency in ethanol and water, respectively (Figure 1 and Table S1). In turn, alcohol–water binary systems—methanol and ethanol mixed with water in a 1:1 ratio—resulted in extracts with the highest polyphenol content. Using methanol–water and ethanol–water mixtures was around three to four times more efficient than pure water. Therefore, ethanol–water (1:1; *v*/*v*) was selected as the safest and most promising solvent, which also aligns with data in the literature [29].

Besides external conditions enabling the extraction of polyphenols, it is worth considering the specific structure–stability relationship facilitating safe separation of the individual group of interest [30]. This is particularly important for anthocyanins, which are unstable at a neutral or alkaline pH. Therefore, different types and concentrations of acids are added to the extraction media, mainly HCl.

The range of HCl percentages referred to in the literature varies from 0.1% to 5%. Higher concentrations provided better results for grapes (3.5%) [31] and even 5% tested on the extraction of polyphenols from barley [32]. The addition of HCl may also decrease viscosity and facilitate the permeation of polyphenols through the cell wall. Due to the possibility of polyphenol degradation at higher temperatures in the presence of HCl, a concentration of 3% was chosen as safe but high enough. As presented in Figure 1, regardless of solvent type, the addition of the acid increased the extraction efficiency for all solvents. The impact is notably visible for pure ethanol and methanol, and seems to have an insignificant influence on the use of pure water. The highest yield of polyphenols was extracted using methanol and ethanol–water (1:1; *v*/*v*) with the addition of acid. Taking into account both solvent type and acid addition, further optimization was carried out using ethanol–water (1:1; *v*/*v*) with the addition of HCl at a concentration of 3%.

#### 2.1.2. Ultrasound Time Exposure

The actual extraction in acidified ethanol–water (1:1; *v*/*v*) was preceded by a short exposure to sonification (up to 15 min) in a water bath.

**Figure 2:**
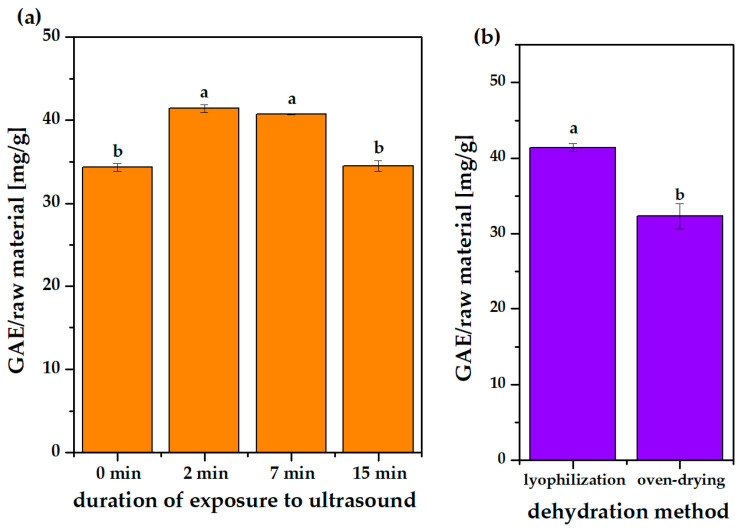
Polyphenol content in raw material extracted from oven-dried grape pomace depending on duration of exposure to ultrasound before extraction in a shaker in acidified EtOH:H_2_O (1:1) at 30 °C, 20 h, l/s 50:1 (**a**) and polyphenol content in raw material extracted considering optimal time exposure to ultrasound, but depending on the dehydration method applied before the whole process (**b**). Different lowercase letters above the bars indicate statistically significant differences (*p* ≤ 0.05) of the means (*n* = 3).

Polyphenolic yield significantly increased from 0 to 2 min (Figure 2a and Table S2), which can be explained by the cavitation phenomenon which facilitates penetration of the solvent into the sample matrix and improves transport between the solid matrix and the liquid phase [33]. Prolonged application of ultrasound may induce the formation of various free radicals, which could react with polyphenols, leading to numerous undesirable reactions such as oxidation, addition, polymerization and decomposition [34]. This apparently happened in our case at a time of 15 min, as the extraction efficiency significantly decreased. As long as there was no significant difference between the polyphenol yield after 2 and 7 min of ultrasound pretreatment, we chose the shorter time for further optimization (time- and energy-effectiveness).

Ultrasound-assisted extraction (UAE) may be as effective as conventional extraction when the latter is conducted at elevated temperatures or for an extended period of time. Thus, the main advantage of UAE is a lower temperature and a shorter time, which may affect the stability of polyphenols [35]. As reported in the literature [28], multiple conventional extraction steps (more than two) do not significantly increase extraction efficiency, while the combination with ultrasound or microwaves is more useful. Therefore, ultrasound-assisted extraction, a straightforward and inexpensive method, appears to be a simple modification of conventional solid–liquid polyphenol extraction that improves its efficiency and economy.

#### 2.1.3. Dehydration Method

Wet grape pomace can undergo various undesired biochemical processes; therefore, we decided that the research material would be thoroughly dried. However, the drying technique and the process conditions influence the chemical composition of the test material. Therefore, in subsequent studies, the efficiency of classical oven-drying and more advanced freeze-drying methods was compared. The data in Figure 2b (Table S3) shows that in the case of oven-drying, the TPC (32.3 mg GAE/g) value was significantly lower than that for the freeze-dried material (41.4 mg GAE/g). Similar conclusions have been reported in the literature [36,37]. Temperature (here, 50 °C in oven-drying) is a factor that not only determines the efficiency of water removal from the material, but also affects the preservation or loss of temperature-sensitive components, such as polyphenols [38]. In freeze-drying, water is removed by sublimation, without heating and therefore damaging the cell structure, which helps preserve valuable components. However, when considering grape pomace processing on a larger scale in terms of protection against unfavorable physicochemical changes and energy savings, one of the solutions may be extraction carried out on the site of pomace collection and on wet material without the drying process. This consideration should be investigated in future studies. In the framework of this study, only dehydrated biomass was tested.

#### 2.1.4. Time and Temperature

Time and temperature are crucial parameters in the extraction process. The optimal biomass dehydration method (lyophilization), solvent composition (ethanol–water, 1:1; *v*/*v* containing 3% of HCl) and ultrasound time application (2 min) were evaluated based on the ability to extract total polyphenols. Time and temperature were optimized by specifying the content of three phenolic groups in the grape pomace: total polyphenol content (TPC), total flavonoid content (TFC) and total anthocyanin content (TAC).

**Figure 3:**
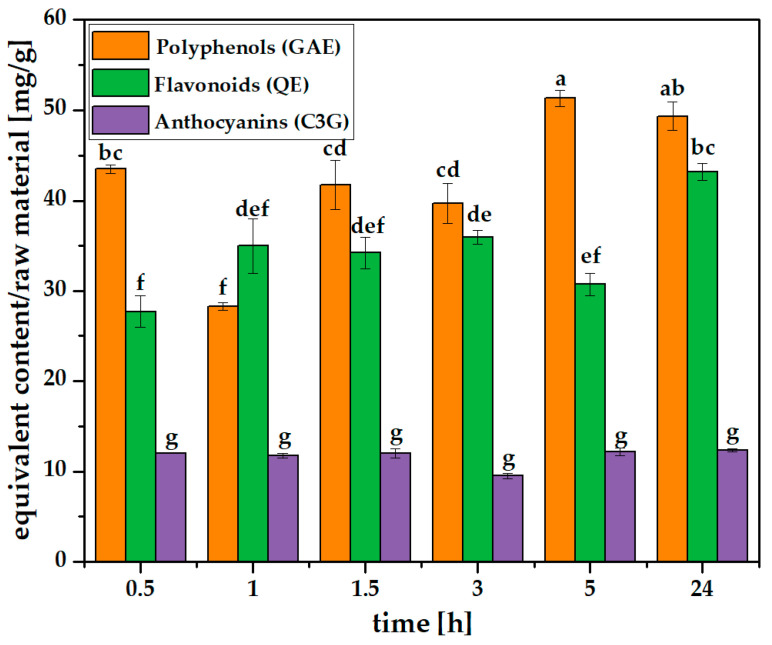
Polyphenols, flavonoids and anthocyanins in raw grape pomace at 30 °C depending on extraction time (0.5–24.0 h). Different lowercase letters above the bars indicate statistically significant differences (*p* ≤ 0.05) of the means (*n* = 3).

**Figure 4:**
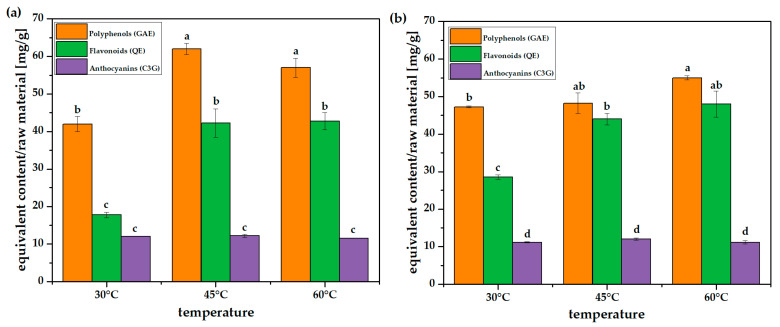
Polyphenol, flavonoid and anthocyanin contents in raw grape pomace mixed in a magnetic stirrer at 30, 45 and 60 °C for 1.5 h (**a**) and 3.0 h (**b**). Different lowercase letters above the bars indicate statistically significant differences (*p* ≤ 0.05) of the means (*n* = 3).

The influence of the extraction duration on process efficiency is presented in Figure 3 (Table S4). After 5 h, the total phenolic content was the highest, and it seems that extending the time up to 24 h is not needed. The content of total flavonoids stayed rather significantly stable in the period of 1–5 h and rapidly increased after 24 h regardless of TPC and TAC, the reason for which is quite unclear, as is that for the significant decrease in TPC after 1 h and the increase after 3 h. The reason might be related to the fact that the evaluated group consists of compounds with different stabilities over time. In turn, the total anthocyanin content was around 10–13 mg C3G/g, irrespective of the extraction conditions, which is clearly visible in the bar graphs in Figure 3 and Figure 4. Anthocyanins are extracted rapidly, regardless of the extraction duration. This may be related to the relatively high acid concentration in the solvent, which facilitates anthocyanin solubility.

Investigation of the impact of temperature on the extraction process was performed for selected temperatures: 30 °C, 45 °C and 60 °C, since the literature mostly reports tests conducted between room temperature (~25–30 °C) and 60 °C [39] and duration times of 1.5 and 3.0 h. Using the same method for material preparation and extraction as in the previous section resulted in an increase in total polyphenol and flavonoid content at 45 °C and 60 °C after 1.5 h, compared to 30 °C. As reported, the increase in temperature enhances the extraction effectiveness because of the higher solubility of the active grape pomace compounds [40]. However, it must be considered that temperature, pH and exposure time influence a variety of polyphenolic compounds differently during extraction [31]. As can be seen, after 1.5 h and 3 h, the highest TPC and TFC values were insignificantly different, and extending the extraction time did not increase their concentration in the extracts (Figure 4, Tables S5 and S6). Similar to previous results, the anthocyanin content was independent of extraction conditions. Conversely, when comparing TPC and TFC values at 30 °C, the influence of time is more evident. The TPC value after 3.0 h reaches the level of TPC at 45 °C after the same duration; however, the flavonoid content is significantly lower. It is worth noting that the “control” extraction at 30 °C shows better repeatability after 1.5 h and 3.0 h than that shown in Figure 3. The method of mixing during extraction may be responsible for improved solvent transfer, as temperature-dependent extractions were performed using a magnetic stirrer. The chosen method of shaking was quite mild; therefore, a longer time might have been needed to achieve uniform recovery of polyphenols. This demonstrates that lower temperatures are sufficient for effective extraction of polyphenols; however, extraction at 30 °C requires longer durations. The selection of time and temperature also depends on the target group of extracted polyphenols. Anthocyanins are easily available at room temperature, whereas the extraction of flavonoids requires a longer duration and a higher temperature, though one not necessarily exceeding 45 °C.

### 2.2. Extraction Using a Binary System: Sub- and Supercritical CO_2_–Ethanol

SC-CO_2_ extraction is one of the most modern methods for obtaining multicomponent mixtures [41]. Carbon dioxide is a non-toxic, inert, non-flammable and low-priced solvent widely used in the food, pharmaceutical and cosmetic industries [42,43,44]. As a non-polar solvent for the extraction of polar polyphenolic compounds, SC-CO_2_ requires the addition of a polar cosolvent, usually ethanol. Sub- or supercritical CO_2_ allows the appropriate pressure to be obtained, as well as rapid mass transfer, and penetration of the cosolvent into the extracted material [45,46].

The addition of a cosolvent to carbon dioxide causes an increase in the system critical temperature compared to pure CO_2_. Based on data from the literature [47], the parameters—temperature and pressure—of the extraction process are designed to obtain both sub- and supercritical conditions. In the case of CO_2_ with 20% ethanol at temperatures of 40 °C and 60 °C, and with 10% ethanol at 40 °C, the extraction was conducted in dense (subcritical) carbon dioxide, while at 60 °C with 10% ethanol the extraction occurred under supercritical conditions. The different conditions influence the polarity of the solvent system and, consequently, the extraction efficiency. The data shown in Table 1 indicates that, at a constant temperature, increasing pressure results in a slight improvement in extraction efficiency. At a temperature of 40 °C, the total phenolic content values range from 6.5 to 7.4 mg/g at 20 MPa and from 7.8 to 7.7 mg/g at 35 MPa, respectively. However, the influence of the ethanol concentration appears to be minimal. At higher temperatures—60 °C—these relationships become unclear. At a higher ethanol concentration of 20%, the highest TPC values were obtained, 10.3 and 11.3 mg/g, suggesting a strong influence of temperature increase on process efficiency. The increase in temperature is accompanied by a decrease in solvent viscosity, leading to improved mass transfer and, consequently, higher extraction efficiency. In turn, at 10% ethanol, the obtained TPC values were the lowest, 4.5 mg/g and 8.6 mg/g.

In the experiments, the highest extraction efficiency (TPC) for the most favorable conditions (Experiment 6, Table 1) was 11.3 mg/g, which is around 30% or less than the results obtained in solid–liquid extraction for the same grape pomace (Figure 4). Similar conclusions were drawn by Chronopoulou et al. The authors compared the extraction efficiency of oleanolic acid from grape pomace using the SC-CO_2_ method (with and without cosolvent) with the solid–liquid method, and showed that the efficiency ratio of the SC-CO_2_ method with a cosolvent (ethanol: 5%, 35 MPa, 50 °C) to the solid–liquid method (5 MPa, 25 °C) was 1:3 [48].

Researchers have predominantly explored grape pomace as a source of polyphenols. But this kind of waste can also serve as extraction material by utilizing SC-CO_2_ with no need to use a cosolvent. Grape pomace provides non-polar compounds like oil containing unsaturated fatty acids possessing high nutritional value. The part of the pomace rich in non-polar compounds is mostly seeds. Other antioxidants in grape oil are vitamin E and phytosterols [49]. Da Porto et al. reported successful extraction of a group of policosanols (long-chain aliphatic alcohols C20–C36) from the wax coating of grape skin and seeds, which might be used in food supplements and cosmetics. The extraction efficiency using SC-CO_2_ for the abovementioned policosanols was nearly the same as that using Soxhlet extraction, but faster and with no need to utilize n-hexane as a solvent [50].

The SC-CO_2_ method is considered a “green” and effective extraction method, but its selection should be preceded by analysis. Supercritical fluid extraction is an efficient and environmentally friendly extraction method and has been widely used in laboratory research and the development of products with high heat sensitivity and added value. However, due to the complex apparatus and high cost of the extraction process, SFE is not widely used for the industrial extraction of plant polyphenols [51]. Non-polar compounds, as a target for extraction from Polish grape waste, seem worthy of investigation in the context of replacing conventional methods with SFE, which is still considered a developing technology.

### 2.3. Liquid-to-Solid Ratio and Time Combination Optimization of Solid–Liquid Extraction Using a Water–Ethanol Binary System

As shown in our experiments, the aim of which was to design a simple and cost-effective method for polyphenol extraction from grape pomace, solid–liquid extraction with a water–ethanolic solution appears to be the most suitable approach. In addition to temperature and time, another important parameter influencing the efficiency of extracting bioactive compounds from plant material is the liquid-to-solid ratio [52,53,54]. Based on preliminary screening experiments, both the liquid-to-solid ratio and time were optimized in ranges from 5:1 to 45:1 mL/g and 180 to 360 min, respectively, using the Response Surface Methodology (RSM) based on a Doehlert experimental matrix [55,56].

Unfortunately, advanced statistical analysis provided rather poor (R^2^ 0.773, adjusted R^2^ 0.395 and *p*-value ≥ 5%) evidence of fitness to the model and the relationship between the two investigated variables (the detailed results, description and methods can be found in the Supplementary Materials).

**Figure 5:**
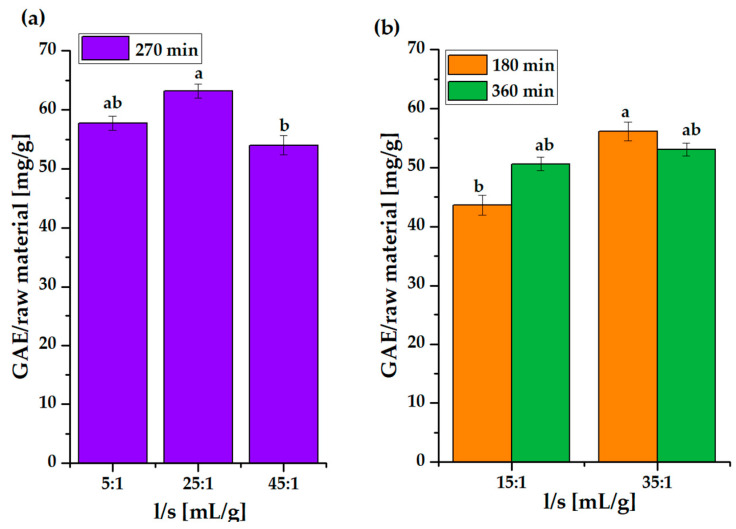
Polyphenol content in raw grape pomace depending on liquid-to-solid ratio for 270 min (**a**) and 180 and 360 min (**b**). Different lowercase letters above the bars indicate statistically significant differences (*p* ≤ 0.05) of the means (*n* = 3).

Regardless of the lack of data-to-model fitness and the relationship of two important parameters, we observed quite a high value of polyphenol yield in one experiment (above 60 mg/g TPC) falling close to the center of the matrix (Figure S1a). It was decided to perform straightforward analysis of the exact combinations of the variables (l/s 5: 1–45:1 mL/g vs. time 160–360) designed for the Doehlert matrix. The results are presented in Figure 5.

Only the combination of a liquid-to-solid ratio of 25:1 mL/g and a duration time of 270 min resulted in a yield above 60 mg/g. Over the same period of time (270 min), lower (5:1) and higher (45:1) liquid-to-solid ratios revealed lower extraction efficiency (Figure S1a).

Increasing the amount of solvent from 5 mL/g allows for better dispersion of individual particles in the solution and improved surface contact, which enhances the extraction of polyphenols [57]. However, a further increase in the liquid-to-solid ratio (45:1) does not result in a further increase in extraction efficiency. Presumably, the lower solvent volume was sufficient to extract all accessible polyphenolic compounds, and additional solvent only resulted in dilution of the extract. Optimizing the amount of solvent added is also important for economic reasons. Excess solvent results in diluted products that require solvent concentration/evaporation in order to obtain an extract in powder form, leading to excessive consumption of the solvent itself, as well as energy.

Over 360 min (Figure 5b), there was no significant difference in polyphenol yield for l/s 15:1 and 35:1. Exceeding the time required for establishing an equilibrium between the polyphenol content in the solution and the biomass carries the risk of oxidation/degradation processes in the extracted compounds due to the presence of oxygen/air in the extraction medium [39]. Over 180 min there was no clear relationship with the previously discussed conditions.

A time of 270 min and a liquid-to-solid ratio of 25:1 mL/g were chosen as the conditions that provided the highest polyphenol yield. A time of 4.5 h is in accordance with the previously discussed optimal duration of extraction.

### 2.4. Polyphenolic Profile of the Extracted Product

The polyphenolic compound extraction conditions influenced not only the extraction efficiency, but also the composition of the obtained extracts [31,58]. Selected extracts obtained at boundary conditions—temperature and time (Table 2)—were analyzed using the HPLC/MS technique. Some phenolic compounds typical of grapes were identified (chromatograms and mass spectra are presented in the Supplementary Materials, in Figures S2–S17). The predominant phenolic acids were protocatechuic acid and *p*-coumaric acid. The identified flavonoids were epicatechin and quercetin, as well as the group of anthocyanins (cyanidin-, delphinidin-, petunidin- and peonidin-glucosides).

Among the non-flavonoid compounds, two phenolic acids were identified. *p*-Coumaric acid belongs to hydroxycinnamic acid derivatives and is more widely distributed in nature than protocatechuic acid, which belongs to the group of hydroxybenzoic acids [59,60]. Phenolic acids, such as *p*-coumaric acid, protect skin against UV radiation as well as free radicals, which makes them suitable active compounds to be used in skin protection agents [61]. Phenolic acids are weak acids, slightly dissociated at a low pH, which has a negative impact on their extraction in polar solvents [62], such as the ethanol–water binary system used in the extraction. On the other hand, a higher temperature facilitates both dissociation and dissolution, enhancing extraction efficiency. This phenomenon is confirmed by the results in Figure 4. A longer extraction time (3.0 h) is beneficial for the content of acids only at a higher temperature (60 °C). Non-flavonoids, however, represent a minority of the extract content (around 10–20%, depending on the extraction conditions). Epicatechin, belonging to flavan-3-ols, is the major component in the examined extracts. However, its content decreases with time and at higher temperatures. Epicatechin may undergo degradation and/or isomerization. These processes are supported by a low pH [40,63], which is confirmed by the results. Epicatechin possesses anti-inflammatory, anti-cancer, anti-diabetic and anti-cardiovascular activity properties [64,65]. Besides epicatechin, another huge group of flavonoids, flavonols, is represented by quercetin. Its content was relatively low in all the examined extracts. Anthocyanins were also identified in the samples. An increase in the extraction temperature up to 60 °C may be harmful for the stability of these compounds (their content decreases by 10–20%) [66,67], which is confirmed by the results in Table S5. However, delphinidin-3-O-glucoside (D3G), one of the most widely distributed glucosides in plants, exhibits the opposite tendency. The content of this particular anthocyanin glucoside in the investigated grape pomace is the highest. It is worth mentioning that it is the most polar anthocyanin out of all the identified compounds in this group (due to the highest number of hydroxyl groups), which enhances its solubility in water and ethanol. Just like other polyphenols, D3G possesses anti-inflammatory and antioxidant properties, but it also has the ability to prevent blood clotting.

These analyses show that the solid–liquid extraction process, although very simple, can also be selective to a certain degree. By controlling its parameters, it is possible to obtain an extract richer in epicatechin or delphinidin-3-O-glucoside. As a result, solutions with various properties, activities and functionalities can be obtained.

The group of polyphenolic compounds that may undergo degradation or autooxidation under acidic pH and elevated temperature are flavonoids. The degradation/autooxidation of flavonols may consequently lead to a decrease in antioxidant activity [68]. At low pH, acid hydrolysis of anthocyanins (a subgroup of flavonoids) occurs: anthocyanin glucosides are hydrolyzed into sugars and an aglycone, forming the flavylium cation, which is stable in an acidic environment and responsible for both the color and antioxidant properties [69]. At higher temperatures, further degradation of these compounds may also occur. For example, Sadilova et al. demonstrated that heating cyanidin-3-glucoside at a high temperature (i.e., 95 °C) in a pH 1 environment first leads to the cleavage of glucose from the anthocyanin molecule, followed by the decomposition of cyanidin into 4-hydroxybenzoic acid and phloroglucinaldehyde, which affects the antioxidant activity of the extract [70]. In the literature, there are also studies showing that the use of low pH and temperatures up to 90 °C allows for the release of a greater amount of polyphenolic compounds from the tested material through hydrolysis and liberation of so-called non-extractable phenolic compounds (associated with cell wall materials), whose presence influences the antioxidant activity of the obtained extract [71,72,73]. The degradation of anthocyanins at high temperatures (higher than the maximum temperature we used—60 °C) is promoted by pH values increasing from slightly acidic to alkaline. This leads to the formation of products such as colorless chalcones, which over time oxidize into brown-colored compounds [74]. In the present study, the amount of anthocyanins obtained was the same in each case. For the entire group of flavonoids, we did not observe a significant decrease in their content under the tested conditions (Figure 3).

Simple solid–liquid extraction, utilizing a green solvent and the assistance of ultrasound, proved to be the most efficient method for obtaining polyphenolic compounds from grape pomace. The research aimed to develop a simple, cost-effective and rapid method for recovering specific compounds from local Polish grape waste following wine production. This method can be implemented using mobile equipment, which can be utilized in many neighboring wineries, as the Polish wine industry is fragmented. Thus, a huge diversity of biomass, a mix of different varieties, requires the initial optimization of polyphenolic compound extraction.

Polyphenol-rich extracts may be applied in food, medicine or cosmetics due to their antioxidant properties. Their encapsulation in liposomes can be an effective strategy for enhancing the delivery of active compounds. Moreover, encapsulation enhances the stability of these compounds, which are easily degradable. One of the extracts containing water-soluble polyphenols was used for encapsulation in order to confirm whether Polish grape pomace could be utilized for liposome formation and serve as an anti-skin agent.

### 2.5. Encapsulation

The polyphenolic extract obtained at 30 °C after 4.5 h of extraction using the ethanol–water binary system was selected for the encapsulation process. Due to the requirements of liposome preparation, the ethanol was evaporated before further processing. Extraction process efficiency was evaluated by the total polyphenol content and anthocyanin content in the grape pomace, 101 ± 8 mg of GAE/g and 12 ± 0 mg of C3G/g of raw material, respectively. As a result, the ethanol-free extract (WE) exhibited TPC and TAC concentrations equal to 8.08 ± 0.64 mg/mL and 0.98 ± 0.00 mg/mL, respectively. The antioxidant capacity of the encapsulated extract expressed as a Trolox equivalent was evaluated using the DPPH assay and was 600 ± 25 µmol TE/g of plant material used. This value is comparable to the TE values reported in the literature [31,75,76], which vary in the order of several hundred micromoles of TE.

**Figure 6:**
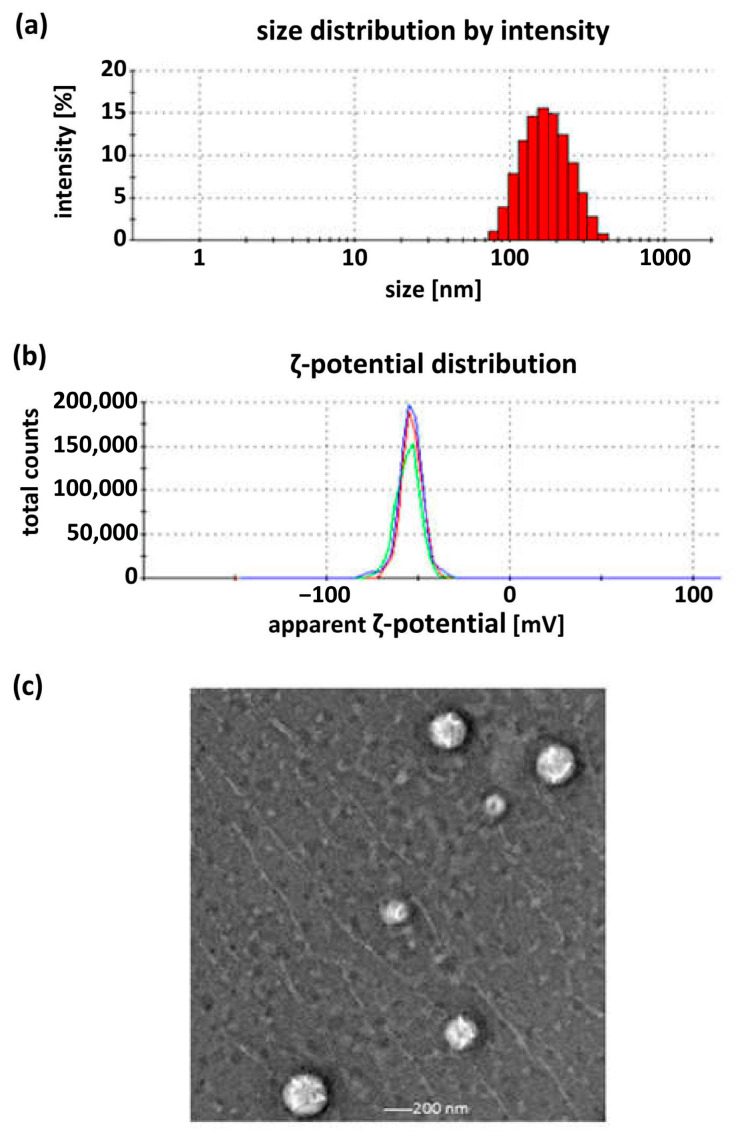
Physicochemical characteristics of the size and morphology of the obtained liposome by means of DLS: size distribution (**a**), ζ-potential distribution (**b**) and TEM (**c**). Example of sample with 2% WE after 7 days.

Polyphenolic extracts are naturally water-soluble; therefore, colloidal structures, liposomes, were selected as carriers for encapsulation. Liposomes are composed of phospholipids with amphipathic properties, featuring hydrophilic head groups and hydrophobic tail regions. These molecules spontaneously assemble into bilayer vesicles or discoidal structures in aqueous environments, forming an internal aqueous compartment. To determine the optimal concentration of the polyphenolic extract (WE), four liposomal systems were prepared with different WE concentrations: 1%, 2%, 5% and 10%, in which the efficiency encapsulation was determined to be 92.88%, 97.81%, 82.67% and 95.75%, respectively. These formulations were characterized in terms of mean hydrodynamic diameter (D_H_), polydispersity index (PdI) and ζ-potential. Measurements were performed using dynamic light scattering (DLS) and electrophoretic light scattering (ELS). For all samples, relatively narrow distributions were obtained for both magnitude and ζ-potential. Transmission electron microscopy (TEM) was used to assess particle size and visualize morphology. The resulting images revealed round objects with sizes similar to those obtained with DLS. Example distributions and images are shown in Figure 6.

**Figure 7:**
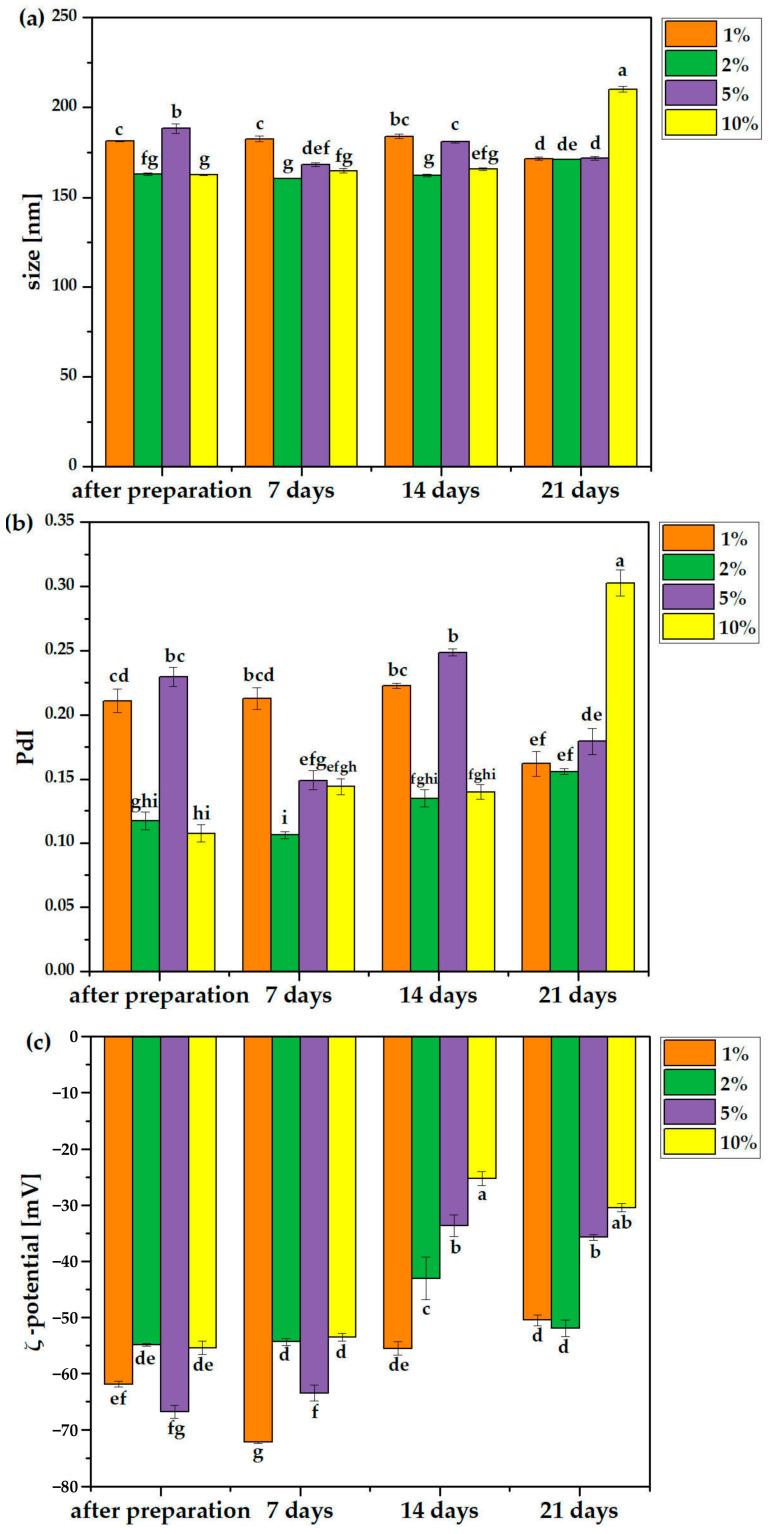
Physicochemical characterization of the obtained liposomes with WE (1%, 2%, 5% and 10%): size [nm] (**a**); polydispersity index (**b**); and ζ-potential [mV] (**c**) after preparation, 7, 14 and 21 days. Different lowercase letters above the bars (**a**,**b**) and below the bars (**c**) indicate statistically significant differences (*p* ≤ 0.05) of the means (*n* = 3).

The physicochemical parameter results are presented in Figure 7, which shows that (a) the size of all liposomes after preparation ranged from about 160 to about 190 nm, regardless of the extract concentration. No significant size changes were observed after 21 days of storage, except in the formulation with the highest extract content (10%), which exceeded 200 nm by the end of the storage period. Similar results were obtained by Chadorshabi et al. [77]. In their study, bioactive compounds extracted from red onion peel were encapsulated in liposomes by freeze-drying, achieving particle sizes ranging from 159.4 to 177.3 nm, ζ-potential values ranging from −38 to −28.6 mV, and improved storage stability up to 21 days. In contrast, encapsulated olive leaf extract in nanoliposomes using mixing and homogenization techniques resulted in particle sizes ranging from 25 to 158 nm and negatively charged surfaces [78]. Significantly larger liposomes were obtained with green tea extract, with a diameter of 419 nm [79]. Fang et al. prepared liposomes loaded with tea catechin using a reverse-phase evaporation method. They demonstrated that the size of the prepared liposomes ranged from 100 to 700 nm [80]. Meanwhile, Lu et al. reported that the average size of green tea polyphenol liposomes was 160.4 nm, and the ultrasonic method allowed for particle size reduction [81]. This result indicates that the liposome particle size depended on the encapsulated bioactive components. This can be explained by hydrogen bonding interactions between the phospholipid polar headgroups and the phenolic compounds of the extracts, as well as hydrophobic interactions between the fatty acid tails of the lipids and the distal hydrophobic regions of the phenolic compounds [82].

Immediately after preparation, the PdI (polydispersity index) values ranged from 0.1 to 0.25, indicating low polydispersity and a homogeneous system. The lowest PdI values were observed in liposomes containing 2% and 10% extract. The PdI values remained relatively stable over the storage period. However, after 21 days, the 10% extract system showed signs of destabilization, which was accompanied by corresponding changes in ζ-potential. Initially, all the formulations exhibited ζ-potential values below –30 mV, indicating high colloidal stability, which is an important index for assessing the surface charges of colloidal systems. Therefore, it is a useful indicator to demonstrate the associations between the encapsulated bioactive materials and the liposome bilayers. The ζ-potential of liposomes containing green tea extract was −59.7 mV [83], while that reported by Dag and Oztop was 35 mV [84], and Lu et al. reported ζ -potentials of nanoliposomes containing green tea polyphenols as −67.2 mV [81]. The high value of the ζ-potential revealed the stability of the suspension, as the charged particles repelled each other and avoided the natural tendency of their aggregation. Liposomes containing negative charges are more stable [85]. Over time, these values increased, suggesting reduced stability. By the end of the storage period, the 1% and 2% extract systems demonstrated the highest stability. Overall, based on all the collected data, the liposomal formulation containing 2% extract exhibited the most favorable characteristics.

Substances encapsulated in liposomes can significantly affect their stability. Depending on the nature of the compound, they can stabilize the lipid bilayer, for example, through hydrophobic interactions or membrane stiffening, or reduce its stability by disrupting lipid organization, increasing membrane fluidity or introducing structural stress. Some compounds can also promote liposome aggregation, particularly when they alter the surface charge, change the balance of repulsive and attractive forces, or affect the hydration layer surrounding the vesicles. The results obtained in this study indicate that WE extracts are effectively encapsulated in liposomes without negatively impacting their stability or aggregation ability. An additional key parameter is encapsulation efficiency, which is influenced by many factors, including the physicochemical properties of the encapsulated substance (such as polarity, molecular weight and solubility), the aqueous-to-lipid ratio and the structural characteristics of the lipid bilayer. Consequently, even when encapsulation efficiency remains similar across formulations, differences in the concentration of the substance or its chemical nature can still lead to differences in liposome stability, which is reflected in parameters such as particle diameter, polydispersity index, zeta potential and susceptibility to fusion or aggregation. Nanoliposomes impregnated with WE extract demonstrated high encapsulation efficiency, good compatibility and satisfactory physicochemical parameters between the extract and the liposomal membrane, supporting the overall stability of the formulation. Such systems are very promising and may be suitable for future applications in both the cosmetics and food industries.

## 3. Materials and Methods

### 3.1. Biomass Source

Biomass source: red grape (*Vitis vinifera* L.) pomace was delivered by a Silesian family winery, ‘Romuald’, Świętej Katarzyny 12, Osiek 59-300, Poland. The waste biomass was collected after press filtration (before the fermentation process). The mix of red grape varieties consisted mostly of Regent and Maréchal Foch.

### 3.2. General Procedure of Solvent Extraction

**Figure 8:**
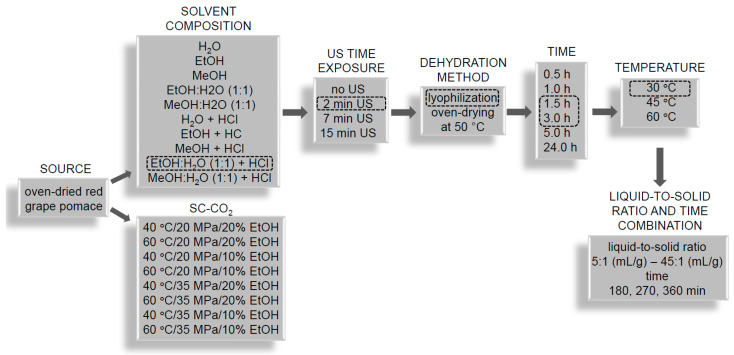
Scheme of extraction optimization steps along with parameters or conditions for red grape pomace used to prepare this paper. Specific parameters circled with a dashed line indicate conditions chosen for the next optimization step.

The biomass was oven-dried in a laboratory dryer (SUP-65M; WAMED, Warsaw, Poland) at 50 °C for 24 h and stored with no light or air access until use. Samples of 0.5–1.0 g of pomace were ground with a pestle in a ceramic mortar, weighed and transferred to flat-bottom flasks. Initial optimization of solvent composition was conducted for a constant period of time (20 h) at 30 °C and with a liquid-to-solid (l/s) ratio of 50:1 mL/g. The sequence of the tested parameters and the optimal parameters chosen at each specific step are presented in Figure 8. A solvent of a certain composition and volume was added, and extraction was performed on a shaker (IKA KS 260 control shaker, IKA, Staufen im Breisgau, Germany) at a moderate speed of 130 rpm. After extraction, the extract was separated from the biomass by filtration through nylon syringe filters (0.22 µm). The filter pore size of 0.22 µm was judged to be more appropriate for polyphenols than a standard filter of size 0.45 µm [86]. The extracts were suitably diluted to fit standard curves prepared for analytical methods evaluating the efficiency of extraction. The tested solvents were as follows: H_2_O, EtOH (Chempur, Piekary Śląskie, Poland), MeOH (Chempur, Piekary Śląskie, Poland), MeOH:H_2_O (1:1; *v*/*v*) and EtOH:H_2_O (1:1; *v*/*v*). The acid added to the extraction media was HCl (Chempur, Piekary Śląskie, Poland) to a final acid concentration of 3%. The impact of ultrasound on the extract was evaluated by exposure of the biomass to ultrasound in a sonication bath (35 kHz; Bandelin Sonorex Digitec DT 106, Bandelin electronics, Berlin, Germany). A part of the biomass was freeze-dried (Alpha 1–4 LSCplus; Martin Christ, Osterode am Harz, Osterode am Harz, Germany). The lyophilization conditions were as follows: product input temperature: −75 °C; shelf temperature: 25 °C; vacuum: 20 Pa; time: 21 h. The time, temperature and solid-to-liquid ratio vs. time combination of the extraction were also optimized, as shown in Figure 8. Extraction was conducted on a magnetic stirrer with heating (IKA C-MAG HS7 digital IKA, Staufen im Breisgau, Germany) or a magnetic stirrer (Electromagnetic stirrer ES 24; WIGO, Pruszków, Poland).

The extract intended for encapsulation was prepared according to the optimized time, temperature, exposure to US and solvent amount, as described above. After extraction and filtration, the ethanol was evaporated using a rotary evaporator. Finally, the water extract (WE) was subjected to liposome preparation.

### 3.3. SC-CO_2_ Extraction

Supercritical CO_2_ extraction of polyphenolic extracts was performed using oven-dried grape pomace. Eight experiments were conducted with specific parameters: temperature at 40 and 60 °C, pressure at 20 and 35 MPa, and cosolvent concentration at 10 and 20%, selected based on the literature [44,46,87,88,89]. Extraction was performed using Multi Vessel-10 Analytical Supercritical Fluid Extraction (Waters) following the standard extraction procedure applied from [90]. Around 5 g of pomace was weighed and transferred to the extraction vessel. There followed a static phase (15 min), a dynamic phase (15 min) and a static phase (15 min) = 1 cycle 4 × 1cycle = extraction time: 3 h. The extrahent (CO_2_ and cosolvent—ethanol) flow rate was 8 mL/min.

### 3.4. Preparation of Liposomes

The liposomes were prepared using the dry lipid film method. In a flask, 500 µL of a chloroform solution of soy lecithin at a concentration of 20 mg/mL and 20 µL was gently mixed, and then the solvent was uniformly evaporated under a warm air stream. The obtained dry film was hydrated with 1 mL of distilled water with dissolved WE at concentrations of 1%, 2%, 5% and 10%, and then squeezed 30 times through a manual extruder with a 100 nm pore size filter applied to ensure identical liposome sizes.

### 3.5. Encapsulation Efficiency

A spectrophotometric method was used to determine the encapsulation efficiency of the EC extract in the liposome suspension. The prepared solutions were measured at 280 nm using a JASCO V-530 UV-Vis spectrometer (Jasco, Tokyo, Japan). The EC emission intensity of the extracts was compared between the tested stock and encapsulated solutions. For this purpose, a 100 μL liposomal aliquot of the EC extract was dissolved in 900 μL of ethanol to disrupt the liposome structure. The encapsulation efficiency was calculated according to the following equation:

(1) $$EE\%\;=\;\frac{amount\;of\;WE\;extract\;in\;liposome\;}{total\;amount\;of\;WE\;extract}\times 100\%$$

### 3.6. Analytical Methods

#### 3.6.1. Determination of Total Polyphenol Content

Total polyphenol content (TPC) was determined using the Folin–Ciocalteu assay [91,92] using gallic acid (Ubichem, Worcestershire, England) as a standard. A test sample of 0.05 mL (suitably diluted in the extraction solvent) was added to 3 mL of distilled water and 0.25 mL of Folin–Ciocalteu reagent (Chempur, Pieksry Śląskie, Poland), mixed and incubated for 5 min at room temperature. Then, 0.75 mL of a saturated sodium carbonate solution was added, mixed and left in the dark for 45 min at room temperature. The absorbance of the sample was measured spectrophotometrically (SPECORD 210; Analytik Jena, Jena, Germany) at 765 nm against a blank. A calibration curve was prepared for the gallic acid, and the TPC results were presented in milligrams of gallic acid equivalent (GAE) per one gram of raw material used.

#### 3.6.2. Determination of Total Flavonoid Content

The total flavonoid content (TFC) in the extract was determined using the aluminum chloride method according to the procedure described by Olędzki et al. [93], using quercetin as a standard. A test sample of 1.0 mL (suitably diluted in the extraction solvent) was added to 4 mL of water and 0.3 mL of 5% sodium nitrite, and incubated for 5 min at room temperature. Then, 0.3 mL of 10% aluminum chloride was added, and the mixture was incubated for 6 min at room temperature. Subsequently, 1 mL of 1 M sodium hydroxide was added to the sample, thoroughly mixed and then incubated for an additional 15 min. The absorbance of the sample was measured spectrophotometrically at 510 nm against a blank. A calibration curve was prepared for the quercetin, and the TFC results were presented in milligrams of quercetin equivalent (QE) per one gram of raw material used.

#### 3.6.3. Determination of Total Anthocyanin Content

Total monomeric anthocyanin content (TAC) was determined using the pH differential method [94,95]. A quantity of 1 mL of the extract was added to 4 mL of potassium chloride buffer at pH 1 and mixed thoroughly. In another test tube, 1 mL of the extract was added to 4 mL of sodium acetate buffer at pH 4.5 and mixed thoroughly. In the case of the extract at pH 4.5, it was sometimes necessary to adjust the pH using NaOH due to the high concentration of HCl in the extract. The absorbance of both solutions was measured at 520 and 700 nm against a blank. The final absorbance value was calculated using the following formula:

(2) $$A\;=\;{\left(A_{520nm}-A_{700nm}\right)}_{pH1.0}-{\left(A_{520nm}-A_{700nm}\right)}_{pH4.5}$$

A calibration curve was prepared for the cyanindin-3-O-glucoside, and the TAC results were presented in milligrams of cyanindin-3-O-glucoside equivalent (C3G) per one gram of raw material used.

#### 3.6.4. Determination of Antioxidant Potential (DPPH)

The antioxidant capacity against DPPH (2,2-diphenyl-1-picrylhydrazyl radical) of the tested extracts was measured as described by Olędzki [93] with modifications. A quantity of 0.050 mL of the test solution (dissolved in methanol to fit the Trolox calibration curve) was added to 1.450 mL of (0.1 mM) methanolic DPPH solution. The mixture was shaken and left in the dark at room temperature for 40 min, after which the absorbance was measured at 517 nm. The antiradical activity was calculated from the calibration curve and expressed as the µM Trolox equivalent (TE) for 1 g of raw material.

#### 3.6.5. HPLC/MS Analysis

HPLC/MS (high-performance liquid chromatography coupled with mass spectrometry) identification of major phenolic compounds was conducted for four selected extracts.

The samples were stored in a freezer (−20 °C) and equilibrated to room temperature before analysis. They were then filtered through a Micro-Spin PES filter (Ciro Manufacturing Corporation, Deerfield Beach, FL, USA). The samples were directly injected into the equipment and were not diluted or concentrated.

The sample extracts (anthocyanins, phenolic acids and flavonoids) were analyzed using a high-performance liquid chromatography (HPLC) system (LC-200; Eksigent, Vaughan, ON, Canada) coupled with a mass spectrometer (QTRAP 5500; AB Sciex, Vaughan, ON, Canada). The HPLC conditions were as follows: column, HALO C18 (2.7 μm particle size, 0.5 × 50 mm; Eksigent); solvent system, solvent A (water/formic acid; 99.05/0.95; *v*/*v*), solvent B (acetonitrile/formic acid; 99.05/0.95, *v*/*v*); and flow rate, 15 μL/min. The elution gradient pattern was as follows: 0–0.5 min (1% B), 0.5–3 min (1–90% B), 3–4 min (90% B), 4–4.2 min (90–1% B) and 4.2–5 min (1% B). The column oven temperature was 45◦C, and the sample injection volume was 5.0 μL. The analyte effluent was connected to an ESI–triple quadrupole–linear ion trap (Q TRAP)–MS instrument. Experiments were set up in negative (phenolic acids and flavonoids) and positive (anthocyanins) ion modes. The ESI source in the positive ion mode operated as follows: the ion spray voltage, temperature, ion source GasI (GSI), GasII (GSII) and curtain gas were set at 5300 V, 350 °C, 35 L/min, 30 L/min and 25 L/min, respectively; the collision gas was set to 20 L/min. The declustering potential, entrance potential, collision energy and collision cell exit potential were set at 100 V, 10 V, 40 V and 15 V. The ESI source in the negative ion mode was the same as in the positive ion mode. Anthocyanins, phenolic acids and flavonoids were identified and quantified by comparing retention times (Rt) and specific parent (Q1) and daughter ion pairs (Q3) using the multiple reaction monitoring (MRM) method, employing data from authentic standards. The calibration curves for the external standards (0.01–15.0 μg/mL) were linear, with coefficients of determination ranging from 0.996 to 0.999.

#### 3.6.6. Characterization and Imaging of Liposomes

The obtained liposomes were characterized by means of size, Pdl, zeta potential and morphology. The liposomes’ average hydrodynamic diameter (DH) and polydispersity index (PdI) were determined by dynamic light scattering (DLS) using a Nano Series Zetasizer from Malvern Instruments, with a detection angle of 173° in optically homogeneous, squared polystyrene cells. All the measurements were performed at 25 °C. Each value was obtained as an average of three runs with at least 10 measurements. The ζ-potential of the nanoparticles was measured by electrophoretic light scattering (ELS) using the same Malvern Zetasizer Nano ZS apparatus. Each value was obtained as an average of three subsequent runs of the instrument, with at least 20 measurements. The DTS (Nano) program was used for data evaluation. The liposomes’ morphology was characterized by transmission electron microscopy (TEM) imaging, performed using an FEI Tecnai G2 XTWIN microscope (FEI, Hillsboro, OR, USA). The samples were prepared by placing a small amount of diluted dispersion on a Cu–Ni grid and stained with 2% uranyl acetate before shooting.

### 3.7. Statistical Analysis

Extractions were performed in triplicate. Averages values and standard errors (SEs) were calculated and presented in bar graphs.

Data was subjected to one-way ANOVA, and mean values were compared by Tukey’s test (*p* ≤ 0.05). Statistical analysis was performed using Origin (Pro), version 2025 (OriginLab Corporation, Northampton, MA, USA). Statistical analysis of the RSM model was provided by NemrodW^®^ Software (Marseille, France), version 2017.

## 4. Conclusions

The wine market is still evolving, but it remains fragmented, especially in regions where viticulture is not widely developed. Our objective was to develop a relatively simple and potentially cost-effective at laboratory scale method for recovering bioactive compounds from Polish wine production waste, with the potential to diversify revenue streams. Due to limited data on the detailed composition of grape pomace from local wineries and the inherent variability of the biomass, preliminary optimization of the extraction process was necessary. The key parameters, including pomace pretreatment methods, solvent type and volume, extraction time and temperature, as well as ultrasonic energy input, were evaluated to obtain relatively high amounts of polyphenols of satisfactory activity. Polish red grape pomace was found to be a rich source of polyphenolic compounds. Under the chosen conditions, the straightforward solvent extraction of the investigated biomass using a shaker yielded at least 30% more polyphenols compared to the more complex and costly supercritical CO_2_ (SC-CO_2_) method, which requires specialized equipment. Moreover, our findings demonstrated that the extraction of specific groups of polyphenols demands tailored conditions. For instance, anthocyanins were extracted efficiently and rapidly, largely independently of extraction parameters, whereas other flavonoids required more precise optimization. The substantial potential of polyphenols, largely due to their antioxidant properties, supports their use in various industrial applications. To enhance their stability and potential usability, the obtained polyphenol-rich extract was encapsulated in liposomes. The results indicate that the encapsulation process was effective and did not compromise the integrity of the active compounds under the conditions tested (21 days). In conclusion, the valorization of grape pomace from local Polish vineyards offers a promising opportunity for developing potentially high-value ingredients that might be used in dietary supplements, food and cosmetic formulations.

## Figures and Tables

**Table 1:** Polyphenol contents in raw material extracted from grape pomace depending on critical parameters using SC-CO_2_ method.

Experiment No.	Temperature	Pressure	EtOH_conc._	^1^ GAE ± SD/^2^ r.m
	[°C]	[MPa]	[%]	[mg/g]
1	40	20	20	7.4 ± 0.12 ^d^
2	60	20	20	10.3 ± 0.06 ^b^
3	40	20	10	6.5 ± 0.21 ^e^
4	60	20	10	4.1 ± 0.04 ^f^
5	40	35	20	7.7 ± 0.13 ^d^
6	60	35	20	11.3 ± 0.03 ^a^
7	40	35	10	7.8 ± 0.11 ^d^
8	60	35	10	8.6 ± 0.33 ^c^

^1^ GAE—gallic acid equivalent, ^2^ r.m.—raw material. Different lowercase indicate statistically significant differences (*p* ≤ 0.05) of the means (*n* = 3).

**Table 2:** Particular major phenolic compounds identified by HPLC/MS in chosen samples extracted in different conditions.

Equivalent/ Standard Name	Structure	Sample			
		1	2	3	4
		Extraction Conditions			
		30 °C 1.5 h HCl	30 °C 3.0 h HCl	60 °C 1.5 h HCl	60 °C 3.0 h HCl
Phenolic acids					
		Concentration in extract [ug/L]			
Protocatechuic acid	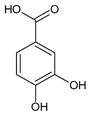	5.2 ± 0.2	3.8 ± 0.0.4	2.8 ± 0.2	4.9 ± 0.0.4
*p*-Coumaric acid	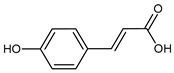	55.2 ± 3.8	45.0 ± 5.0	45.7 ± 1.6	71.0 ± 1.0
Flavonoids					
		Concentration in extract [ug/L]			
Epicatechin	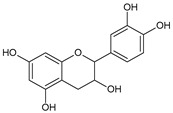	659.7 ± 5.3	422.3 ± 23.6	230.3 ± 9.2	54.2 ± 0.1
Quercetin	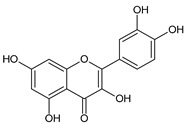	0.2 ± 0.0	0.1 ± 0.0	0.2 ± 0.0	0.5 ± 0.0
Anthocyanins					
		Concentration in extract [ug/L]			
C3G ^1^	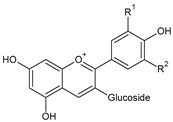 C: R^1^ = OH, R^2^ = H D: R^1^ = OH, R^2^ = OH Pn: R^1^ = OCH_3_, R^2^ = H Pt: R^1^ = OH, R^2^ = OCH_3_	3.2 ± 0.1	2.8 ± 0.1	3.3 ± 0.1	2.5 ± 0.1
D3G ^2^		12.3 ± 0.3	13.1 ± 0.3	87.4 ± 1.6	179.1 ± 2.0
Pn3G ^3^		7.4 ± 1.1	6.3 ± 0.2	4.1 ± 0.1	5.1 ± 0.2
Pt3G ^4^		42.6 ± 2.4	25.3 ± 2.	25.6 ± 2.6	32.1 ± 3.1
		Sum of anthocyanins			
		65.5 ± 3.9	47.5 ± 3.3	120.3 ± 4.3	218.7 ± 5.4

^1^ C3G—cyanidin-3-O-glucoside, ^2^ D3G—delphinidin-3-O-glucoside, ^3^ Pn3G—peonidin-3-O-glucoside, ^4^ Pt3G—petunidin-3-O-glucoside.

## Data Availability

Essential data is contained within the article and the Supplementary Materials.

## Supplementary Materials

The following supporting information can be downloaded at https://www.mdpi.com/article/10.3390/molecules31010072/s1, Table S1: Polyphenol content in raw material extracted from oven-dried grape pomace depending on solvent composition. Extraction conditions: 30 °C, 20 h, l/s 50:1; Table S2: Polyphenol content in raw material extracted from oven-dried grape pomace depending on duration of exposure to ultrasound before extraction in a shaker in acidified EtOH:H_2_O (1:1) at 30 °C, 20 h, l/s 50:1; Table S3: Polyphenol content in raw material extracted considering optimal time exposure to ultrasound, but depending on the dehydration method applied before the whole process; Table S4: Polyphenols, flavonoids and anthocyanins in raw grape pomace at 30 °C depending on extraction time (0.5–24.0 h); Table S5: Polyphenol, flavonoid and anthocyanin contents in raw grape pomace mixed in a magnetic stirrer at 30, 45 and 60 °C for 1.5 h; Table S6: Polyphenol, flavonoid and anthocyanin contents in raw grape pomace mixed in a magnetic stirrer at 30, 45 and 60 °C for 3.0 h; Table S7: Doehlert matrix experimental design for an extraction process for grape pomace with coded (x_i_) and effective variables (u_i_) and values of corresponding experimental and calculated responses of total polyphenol contents (Y_TPC_) in the liquid product fractions; Table S8: Standard deviations of the coefficients of Equation 1 and the coefficient of determination R^2^; Table S9: Analysis of variance of the Doehlert design response; Table S10: Doehlert matrix experimental design for extraction process of grape pomace with coded (x_i_) and effective variables (u_i_); Figure S1: Two (a) and three-dimensional (b) representations of total phenolic content as a function of liquid-to-solid ratio (mL/g) of raw material) and extraction time (min) based on the quadratic polynomial models, Equation 1; Figure S2: Mass spectrum of standard for protocatechuic acid; Figure S3: Mass spectrum of standard for *p*-coumaric acid; Figure S4: Mass spectrum of standard for epicatechin; Figure S5: Mass spectrum of standard for quercetin; Figure S6: Mass spectrum of standard for cyanidin-3-O-glucoside; Figure S7: Mass spectrum of standard for delphinidin-3-O-glucoside; Figure S8: Mass spectrum of standard for peonidin-3-O-glucoside; Figure S9: Mass spectrum of standard for petunidin-3-O-glucoside; Figure S10: Chromatogram of Sample 1 (acids and flavonoids); Figure S11: Chromatogram of Sample 2 (acids and flavonoids); Figure S12: Chromatogram of Sample 3 (acids and flavonoids); Figure S13: Chromatogram of Sample 4 (acids and flavonoids); Figure S14: Chromatogram of Sample 1 (anthocyanins); Figure S15: Chromatogram of Sample 2 (anthocyanins); Figure S16: Chromatogram of Sample 3 (anthocyanins); Figure S17: Chromatogram of Sample 4 (anthocyanins).

## Abbreviations

The following abbreviations are used in this manuscript:

SDG	Sustainable Development Goal
SC-CO_2_	Supercritical CO_2_
RSM	Response Surface Methodology
l/s	Liquid-to-solid ratio
GAE	Gallic acid equivalent
US	Ultrasound
UAE	Ultrasound-assisted extraction
TPC	Total polyphenol content
TFC	Total flavonoid content
TAC	Total anthocyanin content
QE	Quercetin equivalent
C3G	Cyanidin-3-O-glucoside
RSD	Relative standard deviation
SE	Standard error
PEI	Potential Environmental Impact
HPLC	High-performance liquid chromatography
MS	Mass spectrometry
UV	Ultraviolet
D3G	Delphinidin-3-O-glucoside
Pn3G	Peonidin-3-O-glucoside
Pt3G	Petunidin-3-O-glucoside
WE	Water extract
TE	Trolox equivalent
DPPH	2,2-diphenyl-1-picrylhydrazyl
D_H_	Hydrodynamic diameter
PdI	Polydispersity index
DLS	Dynamic light scattering
ELS	Electrophoretic light scattering
TEM	Transmission electron microscopy
ESI	Electrospray ionization
Rt	Retention time
