# Supplementary material for: Design, Sustainable Processing and Nanoliposome Encapsulation of Red Grape Pomace Rich in Polyphenolic Compounds with Antioxidant Activity
Source: Molecules. 2025 Dec 24;31(1):72. doi: 10.3390/molecules31010072 (PMC12786665; doi:10.3390/molecules31010072)
Supplement: Supplementary file 1 [file molecules-31-00072-s001.zip › molecules-3959032-supplementary.pdf]

Supplementary Materials

Design, Sustainable Processing and Nanoliposome Encapsulation of Red Grape Pomace Rich in Polyphenolic Compounds with Antioxidant Activity

Katarzyna Hałdys [1,*], Agnieszka Ciechanowska [1] and Agnieszka Lewińska [2,*]

[1] Department of Chemical Technology, Wroclaw University of Economics and Business, Komandorska 118/120, 53-345 Wroclaw, Poland; agnieszka.ciechanowska@ue.wroc.pl
[2] Faculty of Chemistry, University of Wroclaw, Joliot-Curie 14, 50-383 Wroclaw, Poland
* Correspondence: katarzyna.haldys@ue.wroc.pl (K.H.); agnieszka.lewinska@uwr.edu.pl (A.L.)

Table S1. Polyphenols content in raw material extracted from oven-dried grape pomace depending on solvent composition. Extraction conditions: 30 °C, 20 h, l/s 50:1. Different lowercase indicate statistically significant differences ($p \le 0.05$) of the means (n=3).

solvent type	No acid addition			Addition of 3% HCl		
	TPC			TPC		
	GAE/r.m. (n=3)	SE	RSD	GAE/r.m. (n=3)	SE	RSD
	[mg/g]		[%]	[mg/g]		[%]
H_2O	7.3h	0.6	14.5	8.2h	0.3	7.1
MeOH	20.4f	1.5	12.6	41.2a	1.7	7.1
EtOH	15.4g	0.08	0.9	31.0bc	0.3	1.6
MeOH:H_2O (1:1)	21.8ef	0.2	1.6	25.0de	1.2	8.0
EtOH:H_2O (1:1)	27.2cd	0.7	4.4	34.3b	0.4	2.2

TPC – total phenolic content, GAE, gallic acid equivalent, SE – standard error, RSD – relative standard deviation, r.m. – raw material

Table S2. Polyphenols content in raw material extracted from oven-dried grape pomace depending on duration of exposure to ultrasounds before extraction on the shaker in acidified EtOH:H_2O (1:1) at 30 °C, 20 h, l/s 50:1. Different lowercase indicate statistically significant differences ($p \le 0.05$) of the means (n=3).

Time [min]	TPC		
	GAE/r.m. (n=3)	SE	RSD
	[mg/g]		[%]
0	34.3b	0.4	2.2
2	41.4a	0.5	2.0
7	40.7a	0.06	0.3
15	34.5b	0.6	3.2

TPC – total phenolic content, GAE, gallic acid equivalent, SE – standard error, RSD – relative standard deviation, r.m. – raw material

Table S3. Polyphenols content in raw material extracted considering optimal time exposure to ultrasounds but depending on dehydration method applied before the whole process. Different lowercase indicate statistically significant differences (p ≤ 0.05) of the means (n=3).

Dehydration method	TPC		
	GAE/r.m. (n=3)	SE	RSD
	[mg/g]		[%]
oven-dried	32.3b	1.7	9.1
lyophilized	41.4a	0.5	2.0

TPC – total phenolic content, GAE, gallic acid equivalent, SE – standard error, RSD – relative standard deviation, r.m. – raw material

Table S4. Polyphenols, flavonoids and anthocyanins in raw grape pomace at 30 °C depending on extraction time (0.5-24.0 h). Different lowercase indicate statistically significant differences (p ≤ 0.05) of the means (n=3).

time	TPC			TFC			TAC		
	GAE/r.m. (n=3)	SE	RSD	QE/r.m (n=3)	SE	RSD	C3G/r.m. (n=3)	SE	RSD
[h]	[mg/g]		[%]	[mg/g]		[%]	[mg/g]		[%]
0.5	43.5bc	0.5	1.6	27.8f	1.8	8.9	12.0g	0	0.0
1.0	28.3f	0.4	2.7	35.0def	3	12.1	11.8g	0.25	3.0
1.5	41.8cd	2.8	9.3	34.3def	1.8	7.2	12.0g	0.5	5.9
3.0	39.7cd	2.2	9.6	36.0de	0.8	3.7	9.5g	0.3	5.3
5.0	51.3a	0.9	3.0	30.8ef	1.3	5.7	12.2g	0.4	6.3
24.0	49.3ab	1.6	5.6	43.2bc	0.9	3.7	12.3g	0.2	2.3

TPC – total phenolic content, TFC – total flavonoid content, TAC – total anthocyanin content, GAE, gallic acid equivalent, QE – quercetin equivalent, C3G – cyanidino-3-glucoside equivalent, SE – standard error, RSD – relative standard deviation, r.m. – raw material

Table S5. Polyphenols, flavonoids and anthocyanins content in raw grape pomace mixed on magnetic stirrer at 30, 45 and 60 °C for 1.5 h. Different lowercase indicate statistically significant differences (p ≤ 0.05) of the means (n=3).

temperature	TPC			TFC			TAC		
	GAE/r.m. (n=3)	SE	RSD	QE/r.m (n=3)	SE	RSD	C3G/r.m. (n=3)	SE	RSD
[ºC]	[mg/g]		[%]	[mg/g]		[%]	[mg/g]		[%]
30	42.0b	2.0	6.7	17.8c	0.8	6.0	12.0c	0.0	0.0
45	62.0a	1.5	3.4	42.3b	3.8	12.6	12.2c	0.4	4.6
60	57.0a	2.5	6.2	42.8b	2.3	7.4	11.5c	0.0	0.0

TPC – total phenolic content, TFC – total flavonoid content, TAC – total anthocyanin content, GAE, gallic acid equivalent, QE – quercetin equivalent, C3G – cyanidino-3-glucoside equivalent, SE – standard error, RSD – relative standard deviation, r.m. – raw material

Table S6. Polyphenols, flavonoids and anthocyanins content in raw grape pomace mixed on magnetic stirrer at 30, 45 and 60 °C for 3.0 h**.** Different lowercase indicate statistically significant differences (p ≤ 0.05) of the means (n=3).

temperature	TPC			TFC			TAC		
	GAE/r.m. (n=3)	SE	RSD	QE/r.m (n=3)	SE	RSD	C3G/r.m. (n=3)	SE	RSD
[ºC]	[mg/g]		[%]	[mg/g]		[%]	[mg/g]		[%]
30	47.3b	1.6	0.7	28.5c	1.0	3.5	11.2d	0.2	2.6
45	48.3ab	6.8	8.1	44.0b	2.6	6.0	12.0d	0.3	4.2
60	55a	2.9	1.3	48.0ab	4.2	10.3	11.2 d	0.4	6.8

TPC – total phenolic content, TFC – total flavonoid content, TAC – total anthocyanin content, GAE, gallic acid equivalent, QE – quercetin equivalent, C3G – cyanidino-3-glucoside equivalent, SE – standard error, RSD – relative standard deviation, r.m. – raw material

Liquid-to-solid ratio and time optimization (Doehlert Matrix RSM)

Based on preliminary screening experiments, both the liquid-to-solid ratio and time were optimized in ranges from 5:1 to 45:1 mL/g and 180 to 360 min, respectively, using the Response Surface Methodology (RSM) based on a Doehlert experimental matrix [55,56].

The values of the experimental and calculated responses (total phenolic content yield – Y_{TPC}) are listed in Table S7.

Table S7. Doehlert matrix experimental design for an extraction process of grape pomace with coded (x_i) and effective variables (u_i) and values of corresponding experimental and calculated responses of total polyphenols content (Y_{TPC}) in the liquid product fractions.

**Experiment no.**	**l/s**	**time**	$\mathbf{Y_{exp}}$	$\mathbf{Y_{cal}}$
	**[mL/g]**	**[min]**	**[mg/g]**	**[mg/g]**
1	45:1	270.0	54.00	57.25
2	5:1	270.0	57.50	54.25
3	35:1	360.0	53.60	50.35
4	15:1	180.0	43.70	46.95
5	35:1	180.0	56.70	53.45
6	15:1	360.0	50.60	53.85
7	25:1	270.0	60.50	62.77
7 [1]	25:1	270.0	62.00	62.77
7 [1]	25:1	270.0	65.80	62.77

[1] Experiments repeated at the centre of the experimental region to calculate the standard deviation on the response s_{TPC} = 5.11 for Y_{TPC}.

The results of the polynomial mathematical model were introduced in the quadratic RSM (Equation 2). The final form (Equation 1) is given below.

$$Y = 62.77 + 1.50X_1 + 1.10X_2 - 7.02{X_1}^2 - 13.15{X_2}^2 - 5.77X_1X_2 \quad (1)$$

From the above model, two- and three-dimensional (Figure S8) representations were drawn using NEMROD software of the total polyphenol content (Y_{TPC}) in the raw material, as a function of the liquid-to-solid ratio and time.

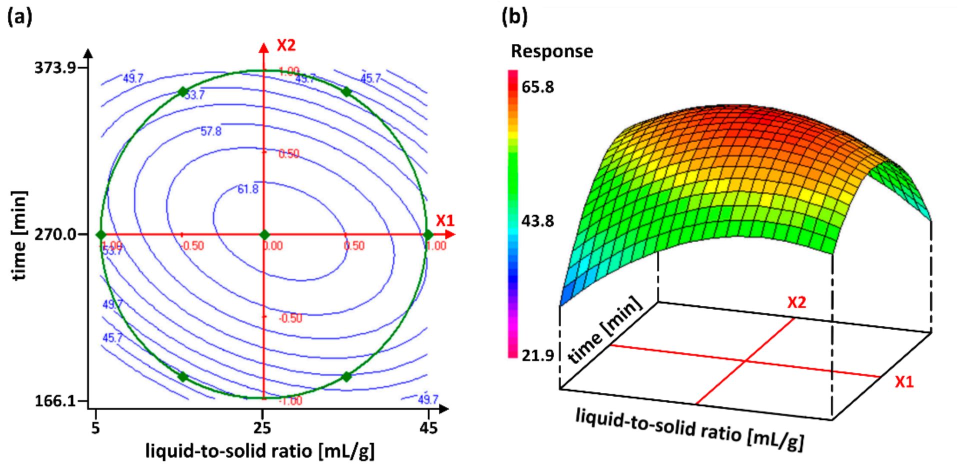

Figure S1. Two (a) and three-dimensional (b) representations of total phenolic content as a function of liquid-to-solid ratio (mL/g) of raw material) and extraction time (min) based on the quadratic polynomial models, equation 1.

Unfortunately, advanced statistical analysis provided rather poor evidence of fitness to the model). The determination coefficient (R^2) was 0.773, which indicates that the model explained 77.3% of the variability and indicating rather unsatisfactory fitness to the data the model was trained on. Validity (p-value) for the model was 0.1 what exceeds our assumed threshold of 5% (p-value 0,05). The adjusted R-squared value was 0.395. The significantly lower adjusted R-squared suggests that there is no relationship between the two investigated variables: liquid-to-solid (X_1) and time (X_2) in the investigated range that can be explained by the obtained equation (coefficients).

Table S8. p-value of the coefficients of equation 1 and coefficient of determination R^2.

Name	Coefficient	p-value
b0	62.77	0.0001
b1	1.50	0.65
b2	1.10	0.73
b11	-7.02	0.23
b22	-13.15	0.07
b12	-5.77	0.40
	R^2 = 0.773	
	R^2 adj. = 0.395	

Table S9. Analysis of variance of the Doehlert design response

Source of variation	Sum of squers	Degrees of freedom	Mean square	Ratio	p-value
Regression	266.92	5	53.38	2.04	0.29
Residuals	78.30	3	26.10		
Validity	63.38	1	63.38	8.49	0.10

Error	14.93	2	7.46
Total	345.22	8	

The Response Surface Methodology (RSM) based on a Doehlert experimental matrix was used to determine two crucial extraction process parameters (independent variables) ensuring optimal yields of total polyphenol content in the raw material (Y_{TPC}). The investigated parameters were: liquid-to-solid ratio (X_1) from 5:1 (mL/g) to 45:1 (mL/g) and time (X_2) from 180 to 360 min (Table 4). The polynomial mathematical model developed for optimisation in this study was a second-degree model

$$Y = b_0 + b_1 \cdot X_1 + b_2 \cdot X_2 + b_{11} \cdot (X_1 \cdot X_1) + b_{22} \cdot (X_2 \cdot X_2) + b_{12} \cdot (X_1 \cdot X_2) \quad (2)$$

where: Y is the experimental response, X_1 and X_2 are the coded experimental levels of the variables, b_0 is the mean value of the response in the central point of the experiment, b_1 and b_2 are the linear coefficients, b_{11} and b_{22} are the quadratic coefficients, and b_{12} is the interaction coefficient.

In this design, each independent effective variable u_i is related to the normalised variable x_i according to the following relation:

$$x_i = \frac{(u_i - u_{i,0})}{\Delta u_i} \quad (3)$$

where x_i varies from –1 to +1, $u_{i,0}$ is the value of the effective variable at the centre of the experimental region (corresponding to $x_i = 0$) and

$$\Delta u_i = \frac{(u_{i,max} - u_{i,min})}{2} \quad (4)$$

is the step with the maximum ($u_{i,max}$) and minimum ($u_{i,min}$) values of effective variable u_i.
The response (Y_{TPC}) was expressed as the total polyphenol content (extraction efficiency) in mg GEA/g of raw material..
A matrix was established to assess the main effect of two factors in 7 runs. In the centre point of the matrix, the experiments were triplicated to check reproducibility and estimate the standard deviation (s_b) of the experimental response. NemrodW® Software (Marseille, France), version 2017, was used for planning the experiments and calculating the coefficients of the polynomial models.

Table S10. Doehlert matrix experimental design for extraction process of grape pomace with coded (x_i) and effective variables (u_i)

Experiment no.	x1	x2	u1	u2
			l/s, [mL/g]	t, [min]
1	1.0000	0.0000	45:1	270.0
2	-1.0000	0.0000	5:1	270.0
3	0.5000	0.8660	35:1	360.0
4	-0.5000	-0.8660	15:1	180.0
5	0.5000	-0.8660	35:1	180.0
6	-0.5000	0.8660	15:1	360.0
7	0.0000	0.0000	25:1	270.0
7 1	0.0000	0.0000	25:1	270.0
7 1	0.0000	0.0000	25:1	270.0

[1] Experiments repeated at the centre of the experimental region

.

Preparation of the extracts according to the Doehlert matrix was conducted as follows: 1 g of lyophilised grape pomace was weighed, and a suitable volume of EtOH:H_2O (1:1, v/v) was added according to the experiment design in Table S10 The extraction medium was then acidified. Before extraction, the flasks containing the pomace and extraction media were exposed to ultrasound for 2 minutes. The time of extraction and the solvent volume at 30 °C were in accordance with the experiment design, as shown in **Table S10.** Doehlert matrix experimental design for extraction process of grape pomace with coded (x_i) and effective variables (u_i)

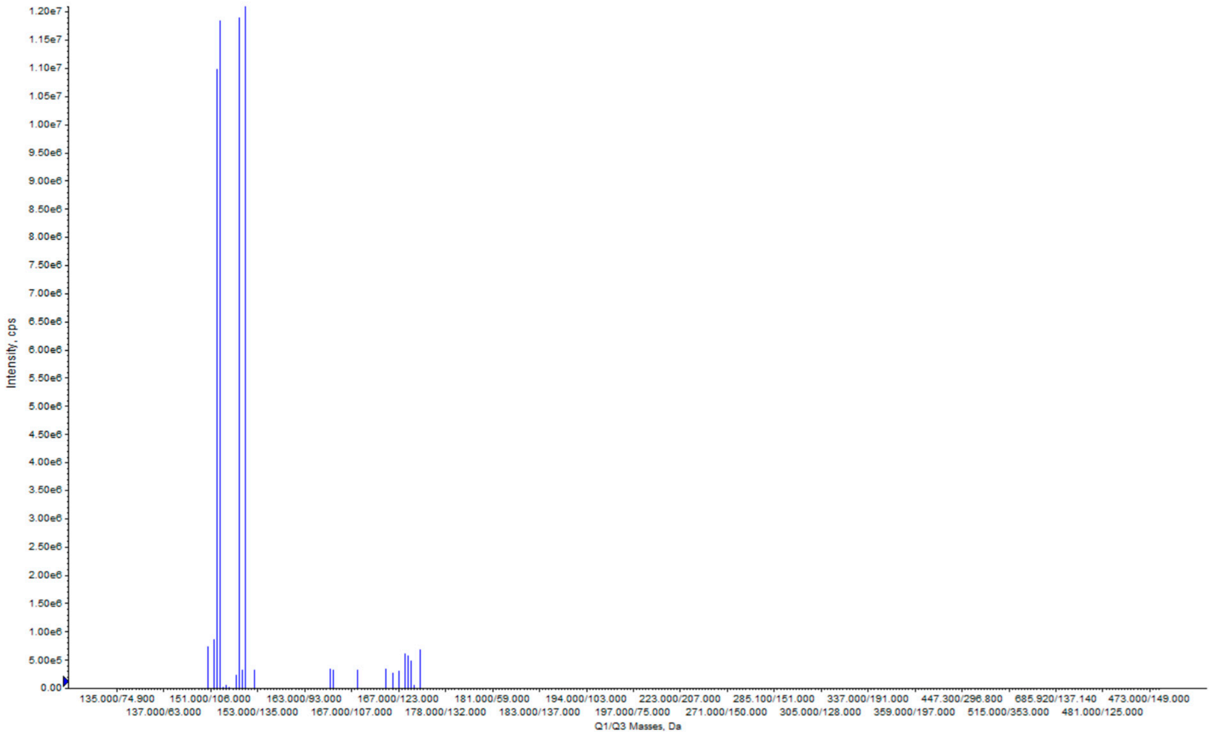

Figure S2. Mass spectrum of standard of protocatechuic acid.

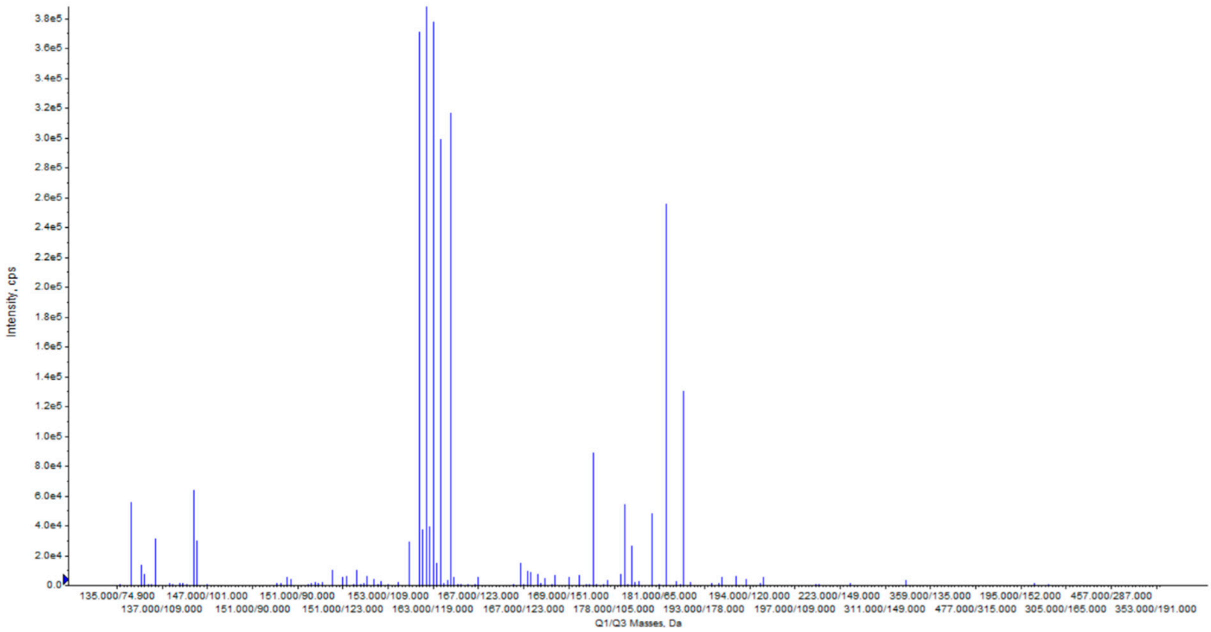

Figure S3. Mass spectrum of standard of *p*-coumaric acid.

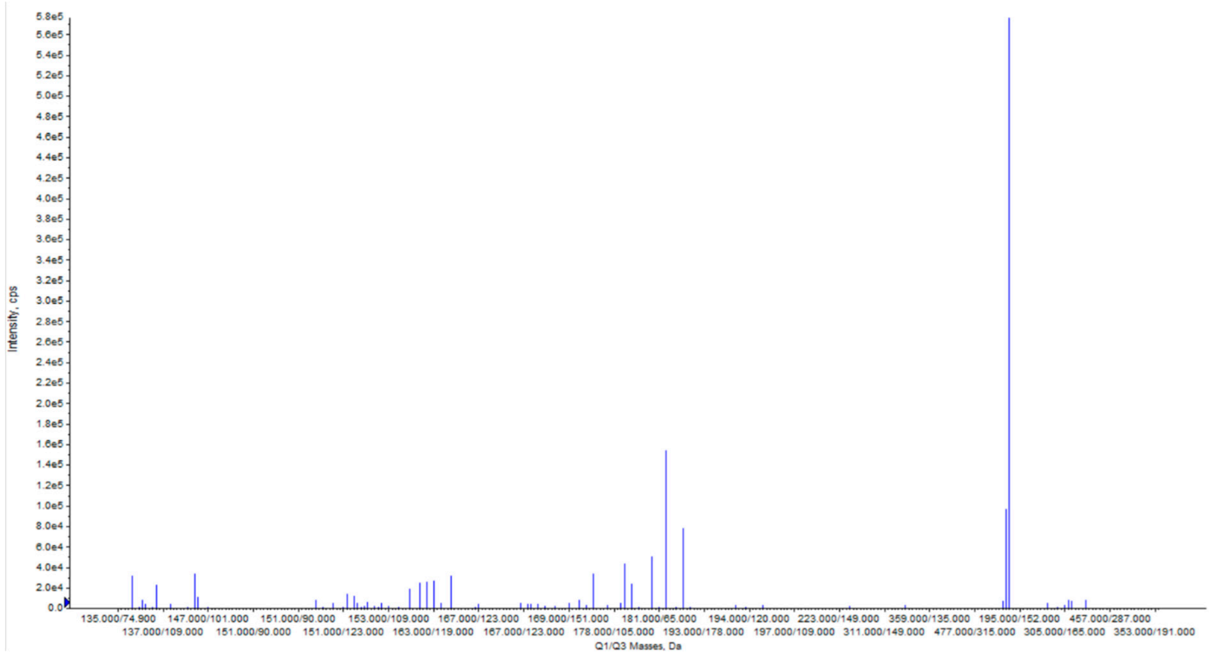

Figure S4. Mass spectrum of standard of epicatechin.

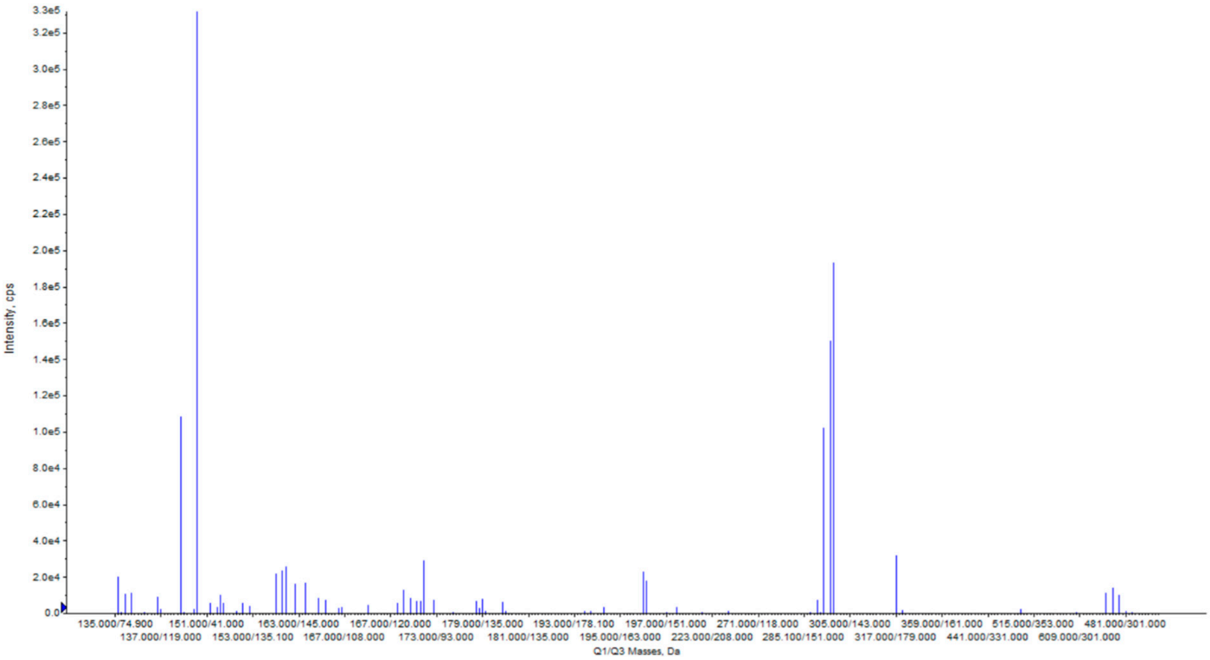

Figure S5. Mass spectrum of standard of quercetin.

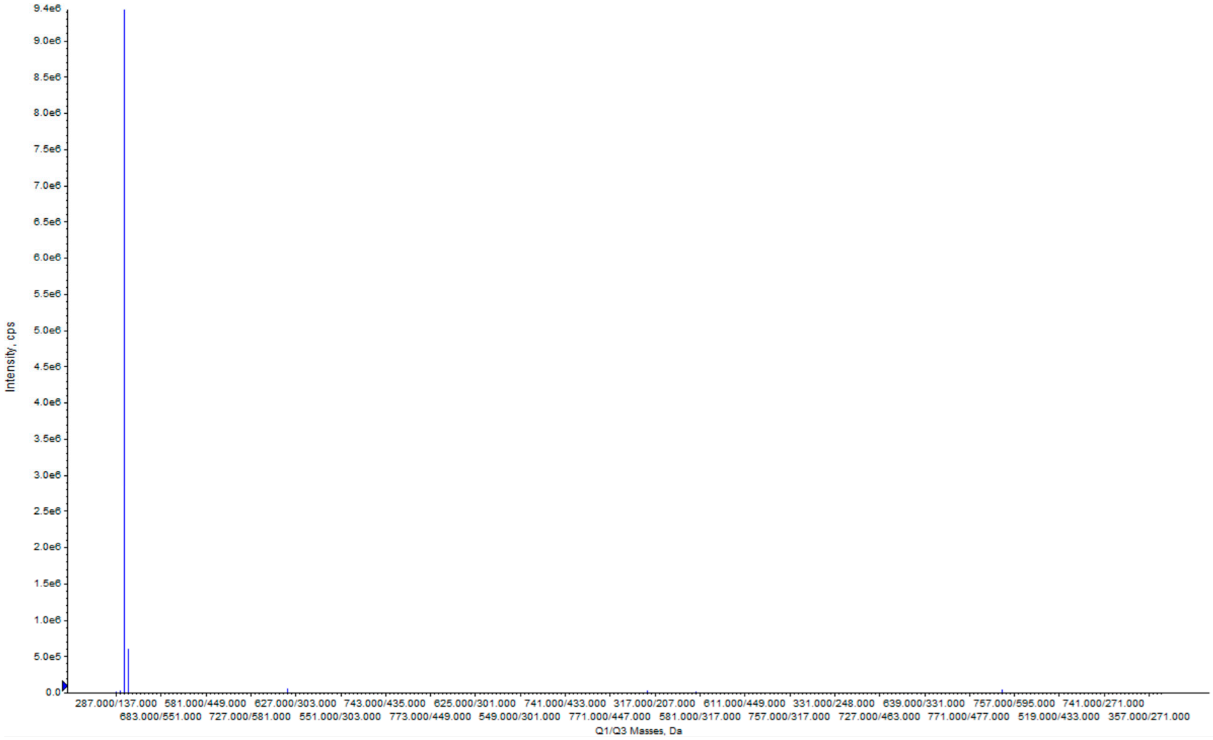

Figure S6. Mass spectrum of standard of cyanidin-3-O-glucoside.

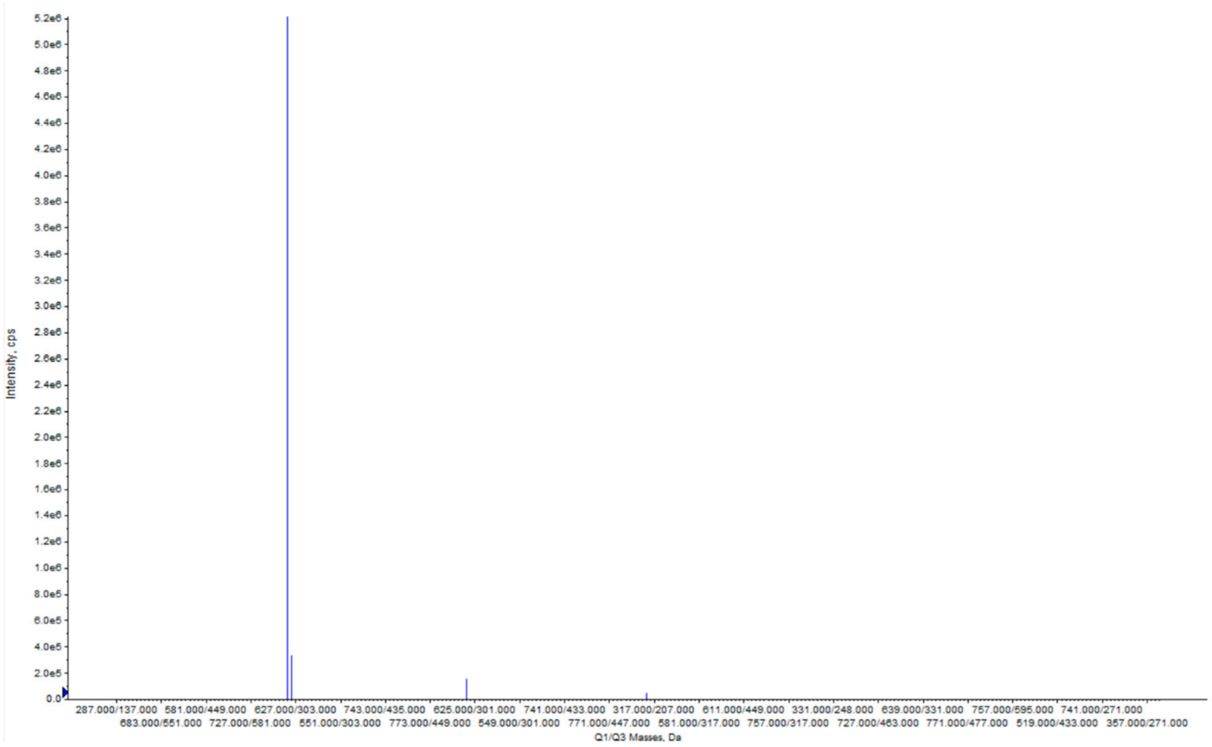

Figure S7. Mass spectrum of standard of delphinidin-3-O-glucoside

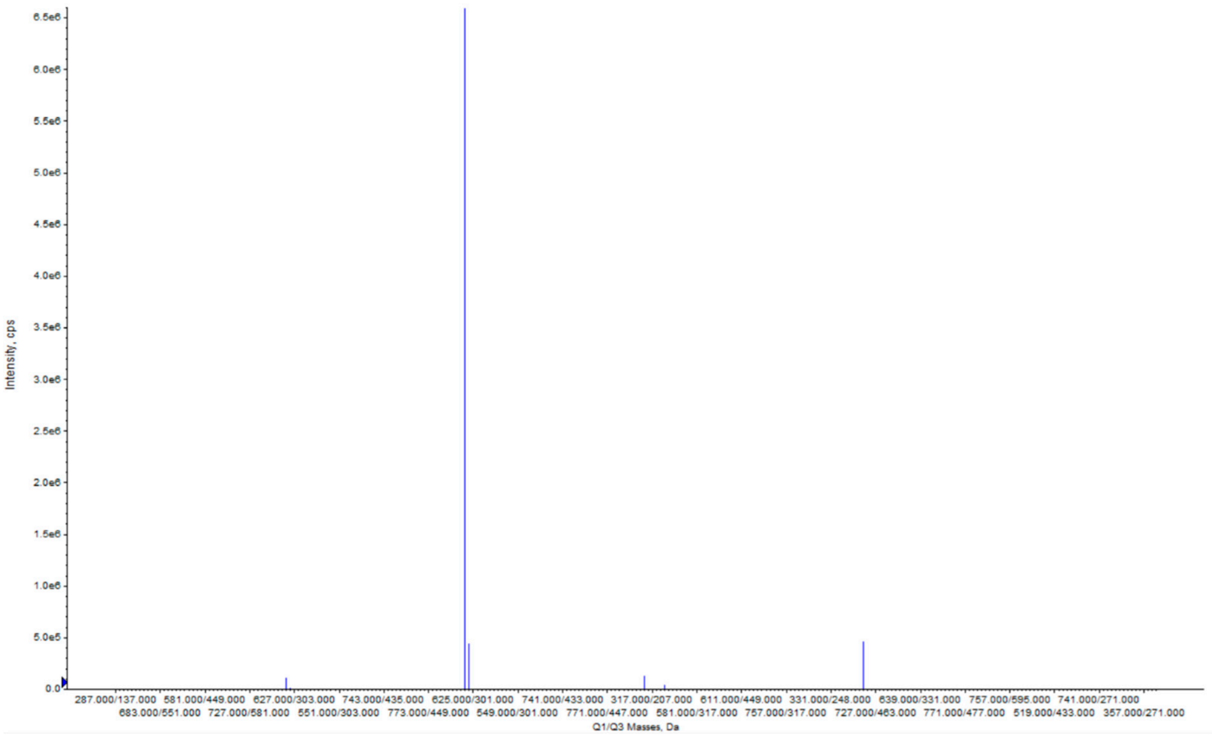

Figure S8. Mass spectrum of standard of peonidin-3-O-glucoside.

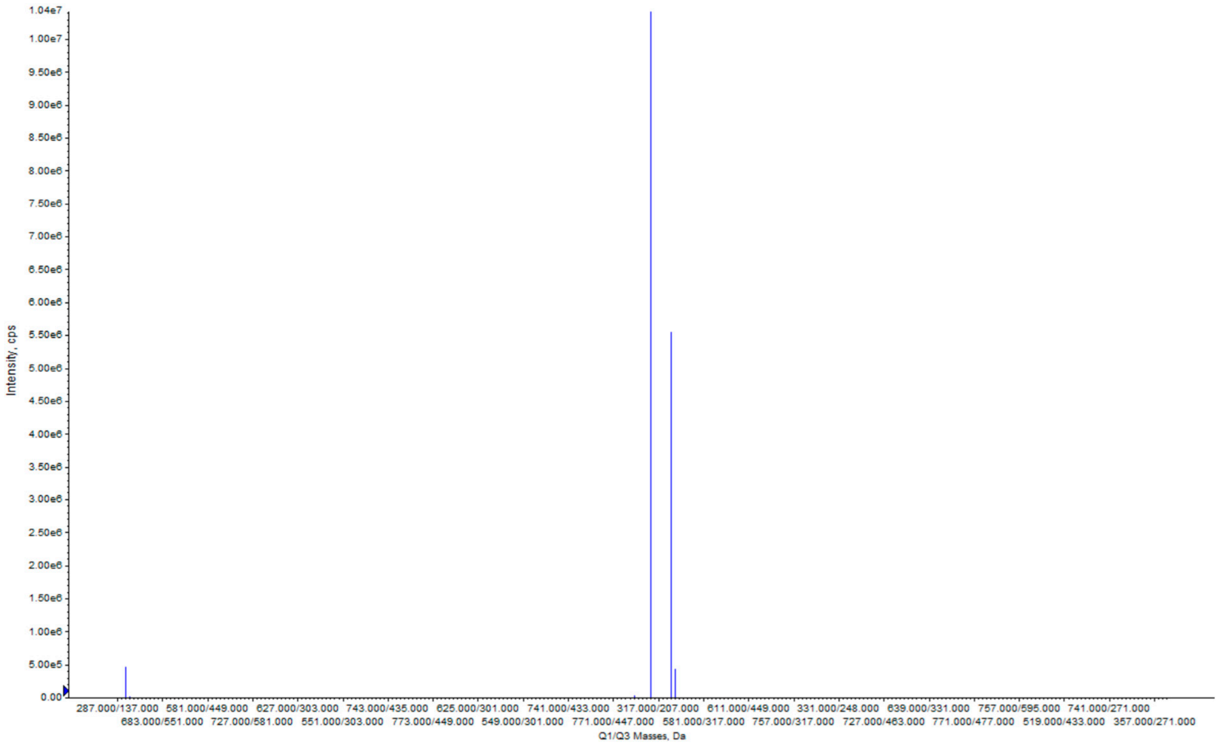

Figure S9. Mass spectrum of standard of petunidin-3-O-glucoside.

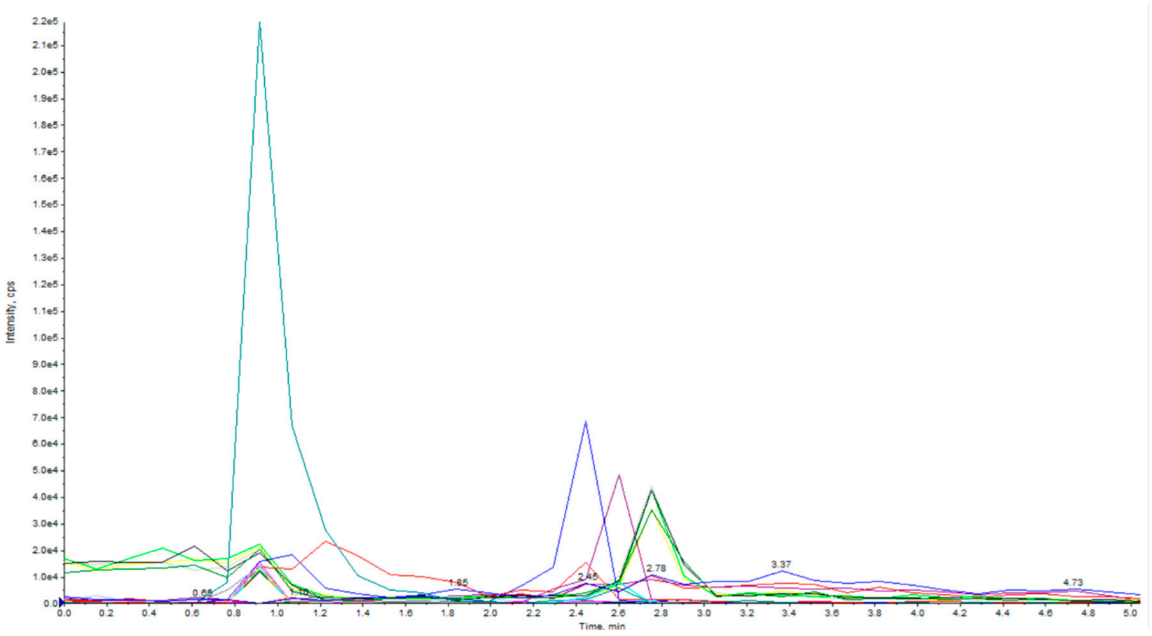

Figure S10. Chromatogram of Sample 1 (acids and flavonoids).

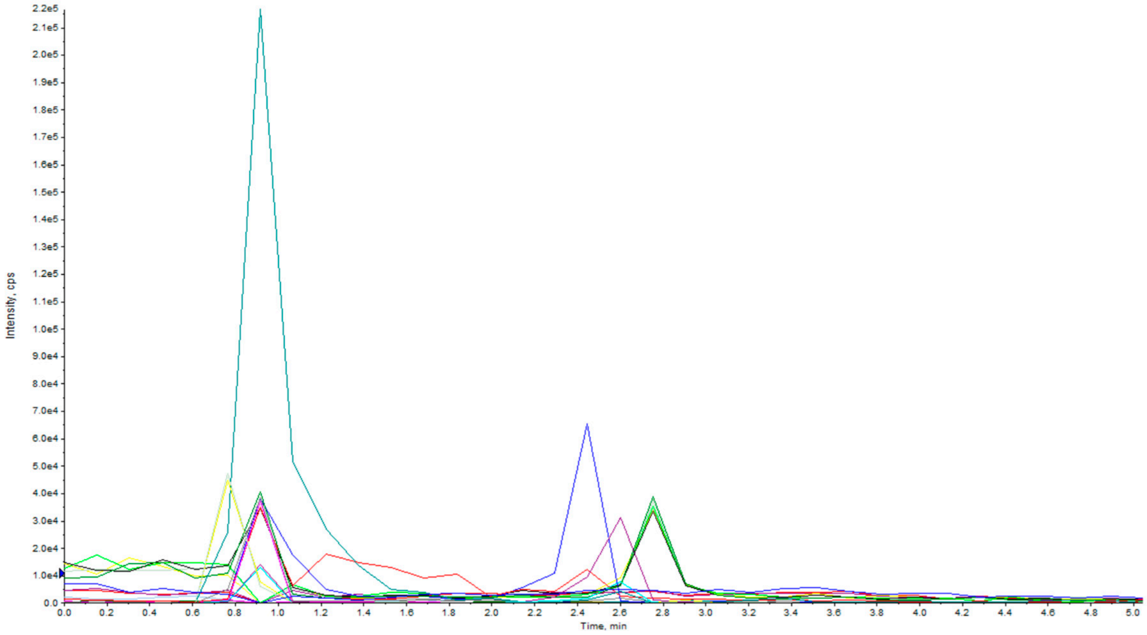

Figure S11. Chromatogram of Sample 2 (acids and flavonoids).

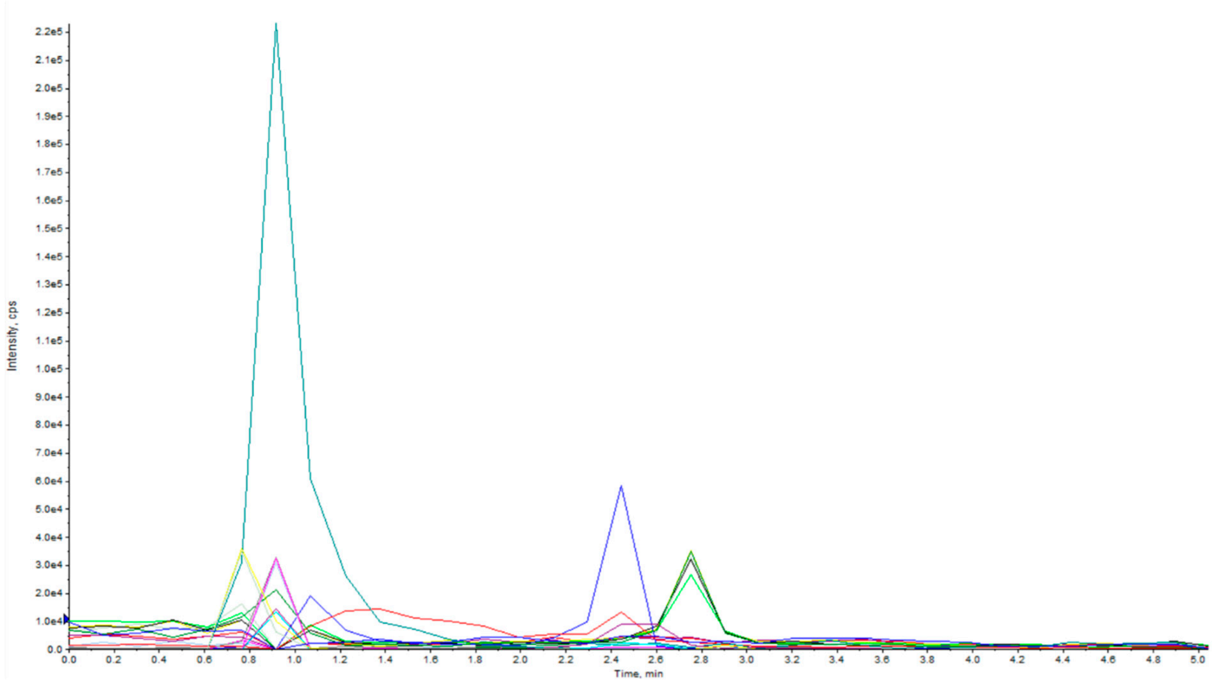

Figure S12. Chromatogram of Sample 3 (acids and flavonoids).

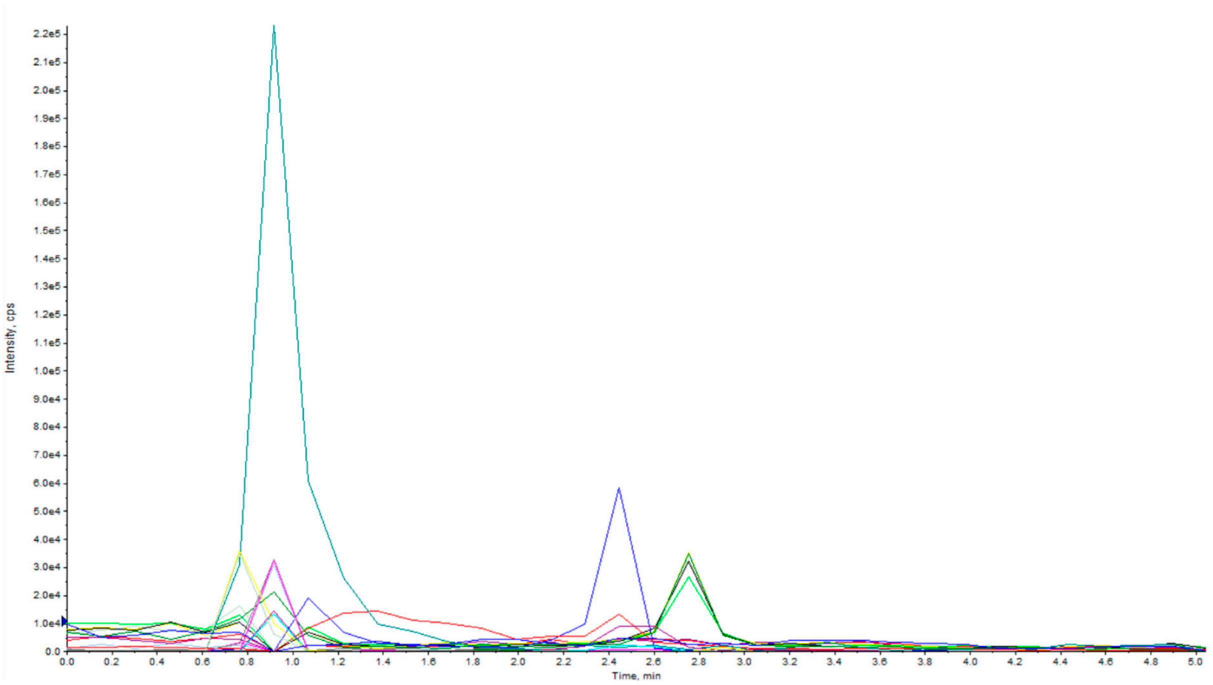

Figure S13. Chromatogram of Sample 4 (acids and flavonoids).

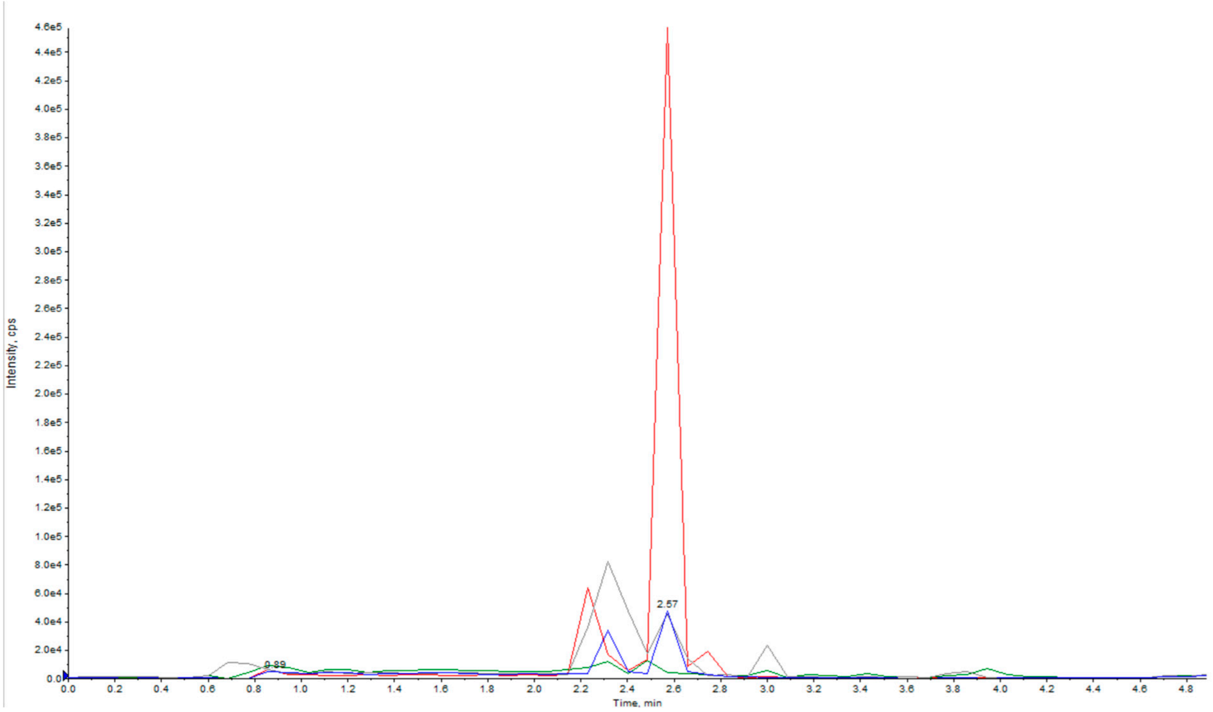

Figure S14. Chromatogram of Sample 1 (anthocyanins)

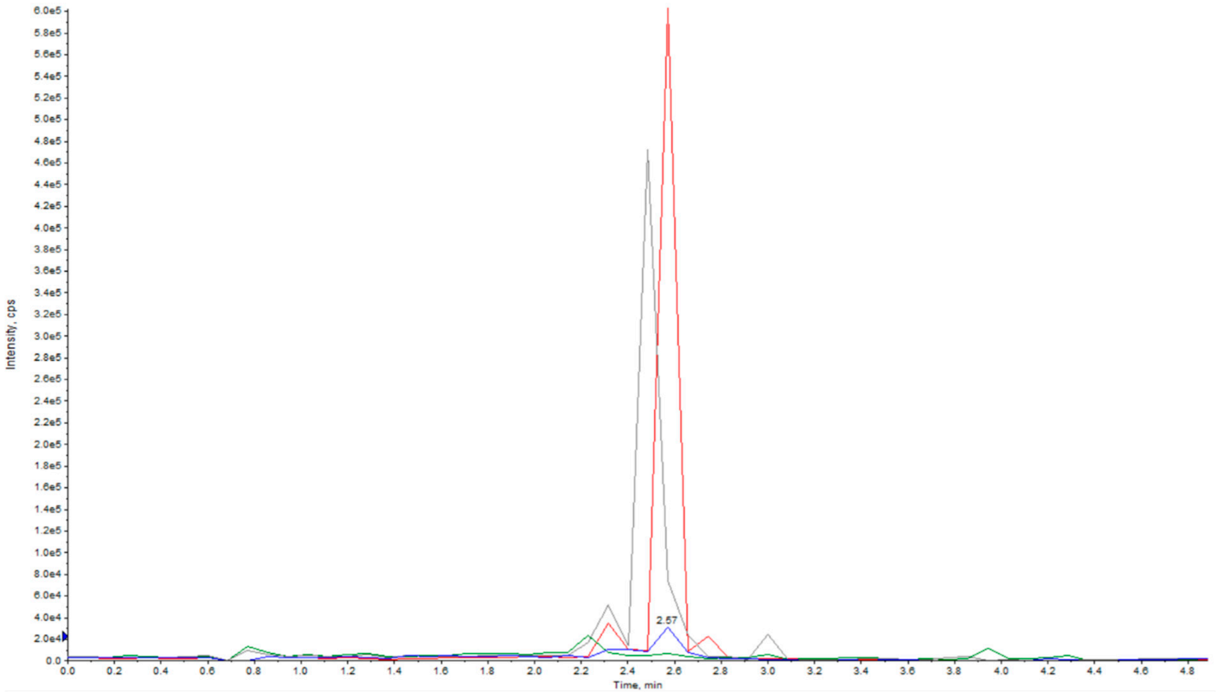

Figure S15. Chromatogram of Sample 2 (anthocyanins).

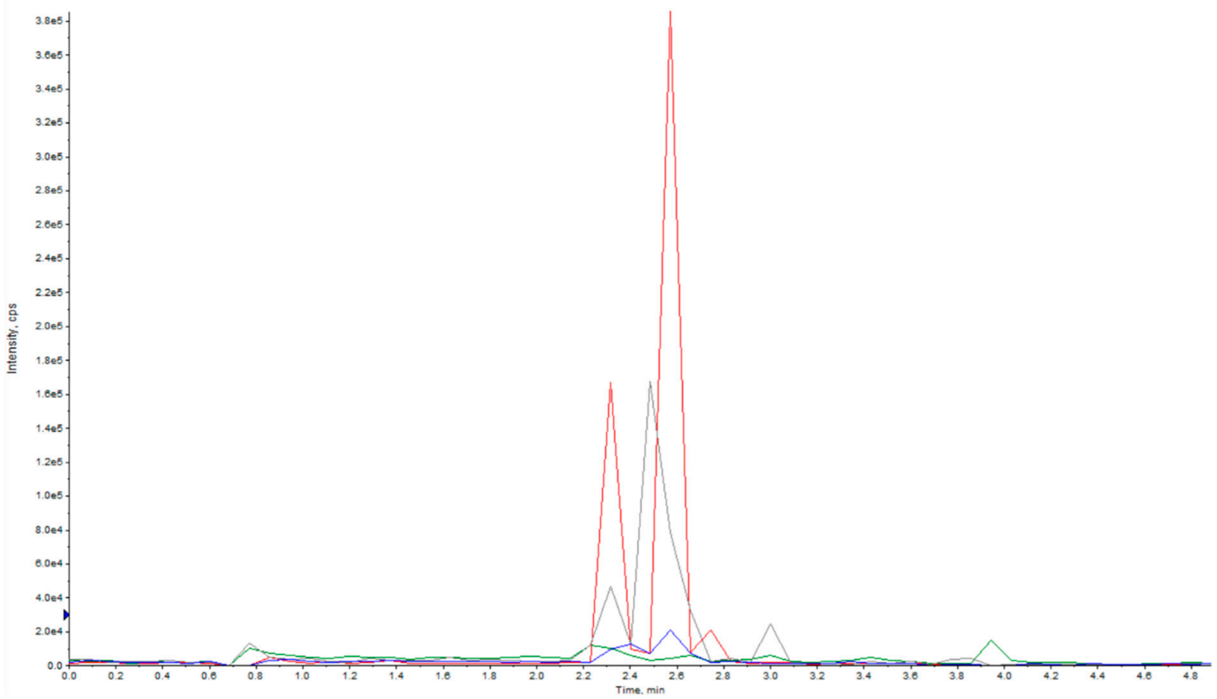

Figure S16. Chromatogram of Sample 3 (anthocyanins).

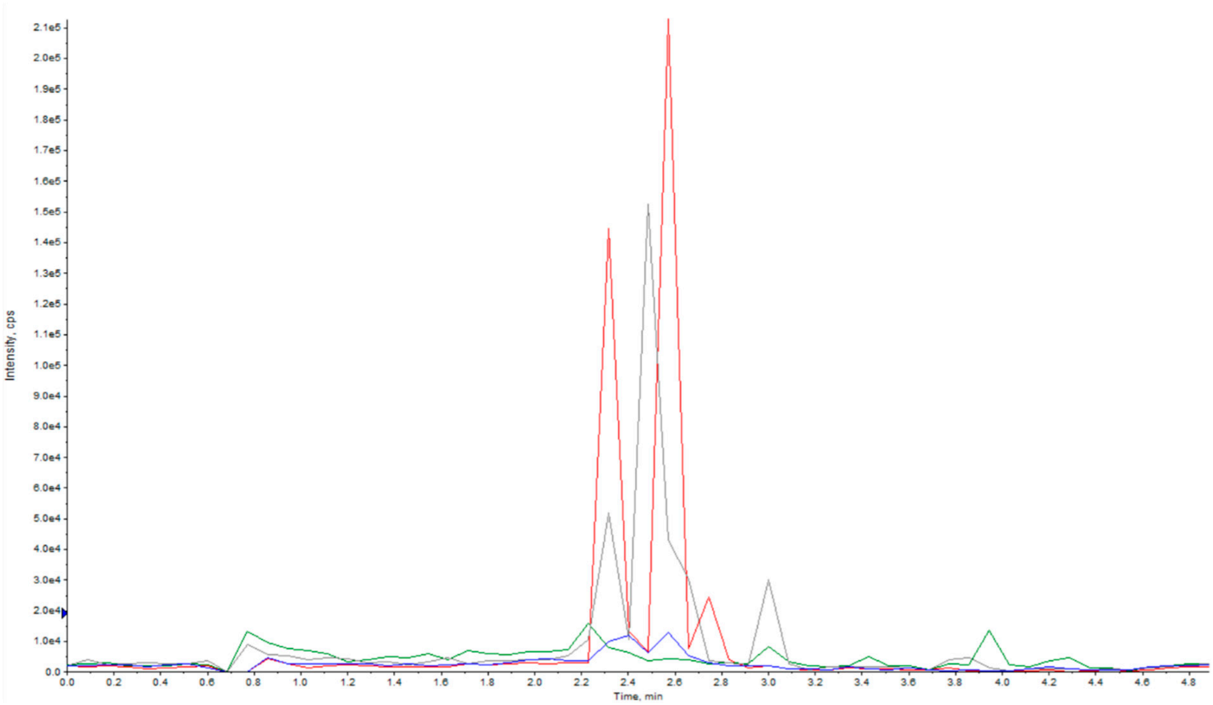

Figure S17. Chromatogram of Sample 4 (anthocyanins).
